# Taro stem-inspired aerogel with vertically ordered channels for high-efficiency solar seawater desalination

**DOI:** 10.1039/d6ra03111k

**Published:** 2026-05-12

**Authors:** Zhuo Wang, Mengya Yu, Jiong Kong, Mi Zheng, Weifeng Li, Yumei Long, Zuoshan Wang

**Affiliations:** a College of Chemistry, Chemical Engineering and Materials Science, Soochow University Suzhou 215123 P.R. China liweifeng@suda.edu.cn yumeilong@suda.edu.cn zuoshanwang@suda.edu.cn; b College of Textile and Clothing Engineering, Soochow University Suzhou 215123 P.R. China

## Abstract

Solar-driven interfacial evaporation has emerged as a promising technology for freshwater production and energy sustainability. It leverages solar energy to efficiently evaporate water, enabling sustainable seawater desalination and wastewater purification. However, designing efficient evaporators that combine rapid water transport and high salt resistance remains a significant challenge. Drawing inspiration from the long-range ordered vasculature and anti-gravity water management mechanisms of taro stem, we fabricated a biomimetic aerogel featuring vertically aligned microchannels through a unidirectional freezing ice templating method. The aerogel integrates a core–shell SiC@C composite as a high-performance photothermal converter, a mechanically robust PVA/PAM double network forming the channel walls, and hydroxyapatite (HA) nanorods serving as both a thermal insulation skeleton and a mechanical reinforcement. In contrast to conventional aerogels with randomly oriented and tortuous pores, the biomimetic honeycomb architectures with vertically aligned channels endow the material with exceptional water transportation, thereby facilitating efficient salt ion diffusion and mitigating salt crystallization. Under 1 sun illumination (1 kW m^−2^), the aerogel achieves a high evaporation rate of 3.24 kg m^−2^ h^−1^, substantially outperforming most reported evaporators with disordered porous structures. The unique vertical channel configuration ensures continuous and stable desalination performance, highlighting its great potential as an effective solution to address global freshwater scarcity.

## Abstract 2

Inspired by taro stems, we have prepared a biomimetic aerogel (SiC@C/HPP), to serve as a highly efficient solar-driven water evaporator.

## Graphical abstract

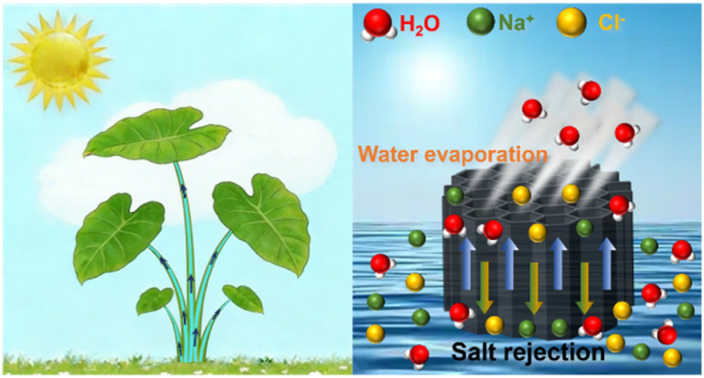

## 1 Introduction

Freshwater is indispensable for life, ecological balance, and socioeconomic development.^1^ Despite covering 70% of Earth's surface, only 3% water on the earth is freshwater and less than 1% is accessible for direct human consumption.^2^ Meanwhile, population growth, climate change, and rapid industrialization are escalating the issue of freshwater scarcity, which poses a global threat to sustainable societies.^3,4^ Consequently, the development of effective technologies to produce freshwater has emerged as a pressing challenge. Given its abundant reserves, seawater desalination has been considered one of the most feasible strategies to address freshwater scarcity. Hitherto, a variety of desalination technologies, including thermal distillation, reverse osmosis, and electrodialysis have been developed for freshwater generation from seawater.^5–7^ Unfortunately, these technologies involve high energy consumption and high cost, limiting their scalable applications.^8,9^ Solar-driven interfacial evaporation (SDIE) technology directly utilizes solar energy to produce clean water from seawater and wastewater, making it a current research focus.^10–13^

Three fundamental attributes contribute to a high-performance solar-driven evaporator. First, photothermal materials must have broad-spectrum absorption and thermal and chemical robustness.^14,15^ Second, the hydraulic architecture should enable rapid capillarity-driven water transport while resisting salt crystallization.^16,17^ Third, thermal localization designs are needed to minimize heat loss.^18,19^ Porous structures play a critical role in solar-driven water evaporation systems by enabling efficient water transport, light absorption, and vapor escape.^20,21^ Aerogels, with their ultrahigh porosity, intrinsic thermal insulation, low density, and large surface area, have emerged as promising materials for solar-driven evaporation systems.^22–24^ Their pore size and configurations can be precisely controlled by freeze-drying route.^25^ The tailored pore size and geometry further regulate vapor diffusion pathways, balancing evaporation kinetics and energy efficiency. Additionally, the networked pore structure provides abundant active sites for loading photothermal compounds, enhancing the stability of the evaporator. Nevertheless, current aerogel-based solar-driven water evaporation systems still face challenges, including low evaporation rate and photo-to-thermal conversion efficiency.^26–28^ In particular, tortuous and disordered pore structures are prone to salt accumulation during solar-driven seawater desalination. This not only severely impedes water transport but also attenuates incident sunlight, impairing evaporation performance and operational durability.^29^ Consequently, rational structural engineering of the evaporator is essential to achieve high water permeability and salt resistance.^22^

Nature, through the evolutionary of natural selection, has engineered well-adapted organizational structures to accomplish intricately biological processes, which provides infinite inspiration for developing new materials and structures with diversity functions. By extracting exquisite designs from nature, many novel materials with special structures have been developed and widely applied.^30,31^ One approach to SDIE biomimetics focuses on fabricating porous structures analogous to those of natural plants by employing traditional preparation methods.^32–34^ The vascular bundles of the taro stems, with their vertically aligned channels and efficient anti-gravity water transport, ensure efficient upward transport of water and nutrients.^35^ Moreover, such hierarchically ordered porous architectures reduce density and increases light absorption by allowing light reflection and multiple-scattering.^36^ These structural features are suitable for the application of solar-driven water evaporation. It is therefore expected that biomimetic aerogels inspired by the taro stem are potential for high-efficiency solar water evaporation.

Drawing inspiration from the unique pore structure of taro stems, this work demonstrates a novel solar-driven water evaporator based on PVA-PAM aerogel incorporating SiC@C nanoparticles and HA nanorods (SiC@C/HA/PVA-PAM) for seawater desalination. The cross-linked PVA-PAM skeleton with vertically aligned channels was fabricated using a unidirectional freezing ice templating method. PVA has excellent film-forming ability and easily forms porous network structures through intermolecular hydrogen bonds.^37,38^ The porous architectures can be further stabilized by crosslinking with PAM, well-known for its high mechanical/chemical resistance.^39–42^ The cross-linked PVA-PAM is rich in hydrophilic groups, which endows the aerogel with strong water absorption capacity, and thereby reduce the enthalpy of water evaporation.^43,44^ Moreover, the porous morphology and polar functional groups of PVA-PAM aerogel provide abundant active sites for loading photothermal compounds through hydrogen bonding or covalent grafting. This synergy enables uniform dispersion of photothermal components and strengthens structural stability. Cubic silicon carbide (β-SiC) has excellent photothermal conversion performance with a bandgap of 2.4 eV, making it suitable for solar-thermal applications.^45,46^ Meanwhile, its high thermal conductivity can facilitate rapid heat dissipation, minimizing energy loss during applications. However, the poor stability of β-SiC limits its further applications.^45,47^ Carbon is one of the most popular materials for solar energy utilization based on its full-spectrum absorption of sunlight and excellent stability.^48,49^ Unfortunately, their high thermal radiation causes conspicuous heat loss and reduces photothermal efficiency.^50^ Carbon composited SiC (SiC@C) not only expands the light absorption range but also maintains the stability of the system.^45,51^ In addition, the introduction of HA nanorods into the PVA-PAM aerogel serve both as support skeleton and thermal insulator.^52–54^ The SiC@C/HA/PVA-PAM (denoted as SiC@C/HPP) composite not only addresses the mechanical fragility typical of conventional aerogels but also leverages its bioinspired vertical channels to enable rapid water delivery and efficient salt rejection. Under 1 sun illumination (1 kW m^−2^), the SiC@C/HPP achieves an evaporation rate of 3.24 kg m^−2^ h^−1^ with a water evaporation efficiency of 90.20%, demonstrating excellent performance in seawater desalination. Moreover, the SiC@C/HPP aerogel exhibits good purification ability, and works well in high-concentration saline and pollutant solutions. Finally, the underlying mechanism for the excellent solar-driven water evaporation of SiC@C/HPP aerogel was systematically discussed.

## 2 Experimental section

### 2.1 Materials

Polyacrylamide (PAM), rhodamine B, cetyltrimethylammonium bromide (CTAB), polyvinyl alcohol (PVA), sodium chloride (NaCl), and glutaraldehyde (50%) were purchased from Shanghai Aladdin Biochemical Technology Company. Oleic acid, sodium dihydrogen phosphate dihydrate (Na_2_H_2_PO_4_ 2H_2_O), silicon carbide (SiC) were purchased from Shanghai McLean Biochemical Technology Company. Calcium nitrate (Ca(NO_3_)_2_), anhydrous glucose, sodium hydroxide (NaOH) were purchased from Sinopharm Chemical Reagent Company. Methanol was purchased from Shanghai Yi En Chemical Technology Company. Acetic acid (glacial acetic acid) was purchased from Chinasun Specialty Products Company. All chemicals were used as received without further purification. Deionized water was used for all relevant experiments.

### 2.2 Preparation of SiC@C composite material

First, 0.05 g SiC, 3.6 g glucose, and 70 ml deionized water were mixed in a beaker and stirred for 30 minutes. Subsequently, 3.6 g CTAB was added to the uniformly dispersed suspension, followed by another 30 minutes of stirring. The mixture was transferred into a 100 ml PTFE lined vessel, which was sealed in a steel autoclave. After heating at 180 °C for 16 hours in an oven, the resulting product was centrifuged at 8000 rpm for 10 minutes. Finally, the collected precipitate was dried in a 60 °C oven for 24 hours to obtain the SiC@C composite material.

### 2.3 Preparation of hydroxyapatite (HA) nanorods

Hydroxyapatite HA nanorods were synthesized using a hydrothermal method.^55^ Firstly, 15.0 ml of anhydrous ethanol, 1.5 g of NaOH and 7.5 g of oleic acid were placed in a beaker, followed by the addition of 20.0 ml of Ca(NO_3_)_2_ solution (2.1 mol L^−1^), and the mixture was stirred for 30 minutes. Subsequently, 20.0 ml of Na_2_H_2_PO_4_ solution (concentration 1.26 mol L^−1^) was added, and the mixture was stirred for 1 hour. The resulting white mixture was transferred to a 100 ml PTFE lined vessel, which was sealed within a steel autoclave. After heating in an oven at 120 °C for 8 hours, the solution in the PTFE lined vessel was centrifuged at 8000 rpm for 6 minutes. The collected product was washed three times alternately with deionised water and ethanol, ultimately yielding hydroxyapatite (HA) nanorods.

### 2.4 Preparation of biomimetic SiC@C/HA/PVA/PAM (SiC@C/HPP) aerogel

First, 0.1 g of SiC@C nanomaterial and 0.3 g of hydroxyapatite (HA) nanorods were dispersed in 25 mL of deionized water in a beaker, followed by stirring for 30 minutes. Then, 6.0 g of PVA aqueous solution (0.1 g ml^−1^) and 2.5 g of PAM aqueous solution (0.03 g ml^−1^) were added to the mixture, and stirring continued for another 30 minutes. While stirring, 250 µL of glacial acetic acid and 40 µL of glutaraldehyde (50%) were slowly introduced, and the mixture was stirred for an additional 1 hour. Finally, the homogeneous suspension was poured into a silicone mold with 2 cm side length. The mold was placed on a copper block partially immersed in liquid nitrogen. As the liquid nitrogen evaporated, the copper block remained cold, enabling directional control of ice crystal growth inside the mold. After the sample was completely frozen, it was freeze-dried using a freeze-dryer (SCIENTZ-10 N) and then thermally crosslinked at 80 °C for 1 hour. This process yielded the biomimetic SiC@C/HPP aerogel. To evaluate the benefits of the vertical channel structure, a control sample with a disordered pore architecture was prepared using the same procedure, except that unidirectional freezing was replaced by freezing at −20 °C for 12 hours in a conventional freezer, followed by identical freeze-drying and thermal crosslinking steps.

### 2.5 Characterization

SEM micrographs were acquired by a field emission scanning electron microscope (Regulus 8230, Hitachi, Japan). TEM micrographs were obtained by a field emission transmission electron microscope (HT7700, Hitachi, Japan). XRD tests were performed by X-ray Powder diffractometers (D8 Advance, Bruker, Germany). FTIR tests were performed by a Fourier transform infrared spectrometer + FT-IR Microscopes (VERTEX 70+HYPERION 2000, Bruker, Germany). The compressive mechanical properties were measured by a universal testing machine (Drick, China) with a displacement rate of 10 mm min^−1^. An UV-VIS-NIR spectrophotometer with an integrating sphere (UV-3600, Shimadzu, Japan) was applied to measure the light absorption and reflectivity from 200 ∼ 2500 nm. Metal ion concentrations were measured using Inductively Coupled Plasma Optical Emission Spectroscopy (ICPOES) (5110, Agilent, America).

### 2.6 Solar water evaporation, seawater desalination, and water purification experiments

In the solar-driven water evaporation experiment, a xenon lamp (CEL-S500, CEAULIGHT) was used to simulate sunlight. The solar irradiance was calibrated using a fully automatic photometer (CEL-NP2000-2 A, CEAULIGHT). During testing, a 25 ml beaker was filled with the liquid. The aerogel sample was fixed above the beaker using an ethylene vinyl acetate copolymer foam board, with its base immersed in water and its top surface exposed to air. Mass changes during the solar evaporation of pure water or seawater were recorded in real time using an electronic balance (TE124S, Sartorius, Germany). Surface temperatures were monitored with an infrared thermal camera (FOTRIC 246 M, CEAULIGHT), which was connected to a computer for data acquisition. Based on these measurements, the water evaporation rate of the solar-driven water evaporator (SiC@C/HPP aerogel) can be expressed in terms of the change in water mass per unit time, calculated using the following formula:

1 *v* = Δ*m*/(*S* × Δ*t*)

where Δ*m* represents the change in the mass of water during evaporation (kg), *S* denotes the upper surface area of the water evaporator exposed to light, and Δ*t* denotes the duration of illumination.

The water evaporation efficiency (*η*) of the biomimetic SiC@C/HPP aerogel was calculated using the following equation:^56^

2 *η* = (Δ*ν* × (*L*_V_ + *Q*))/*P*_in_

Here, Δ*ν* represents the water evaporation rate of the SiC@C/HPP aerogel under standard solar radiation (1 kW m^−2^), calculated as the difference between the evaporation rate under illumination and the evaporation rate in the dark; *L*_V_ is the latent heat of phase change of water; *Q* is the sensible heat of phase change per unit mass of water; and *P*_in_ is the input solar energy. The formula for the sensible heat of phase change of water is:

3 *Q* = *C* × (*T* − *T*_0_)

where *C* denotes the specific heat capacity of water (4.18 J g^−1^ K^−1^), *T*_0_ the initial water temperature, and *T* the steady-state evaporation temperature measured at the air-liquid interface.

For desalination assessments, natural seawater samples were collected from the Bohai Sea (40.6°N, 120.8°E). Simulated wastewater for purification studies was formulated by dissolving NiCl_2_ 6H_2_O, PbCl_2_, CrCl_3_ 6H_2_O and MnCl_2_ 4H_2_O in deionized water, yielding solutions containing Ni^2+^, Pb^2+^, Cr^3+^ and Mn^2+^ ions at 100 mg L^−1^ each. Rhodamine B contaminant solutions were prepared at 10 mg L^−1^, used as the simulated effluent.

## 3 Results and discussion

High-performance solar evaporators must exhibit exceptional water transport and salt tolerance. As a typical aquatic plant, the taro stem has developed a highly efficient transport system. Inside the stem, well-ordered vascular bundles allow water and nutrients to move quickly toward the leaves at the top.^56^ In addition, the vascular walls contain abundant hydrophilic substances, which greatly reduce resistance to water flow through the channels. Together, the vertical channels and the hydrophilic inner walls give the taro stem a remarkable capacity for rapid water transport.

**Fig. 1:**
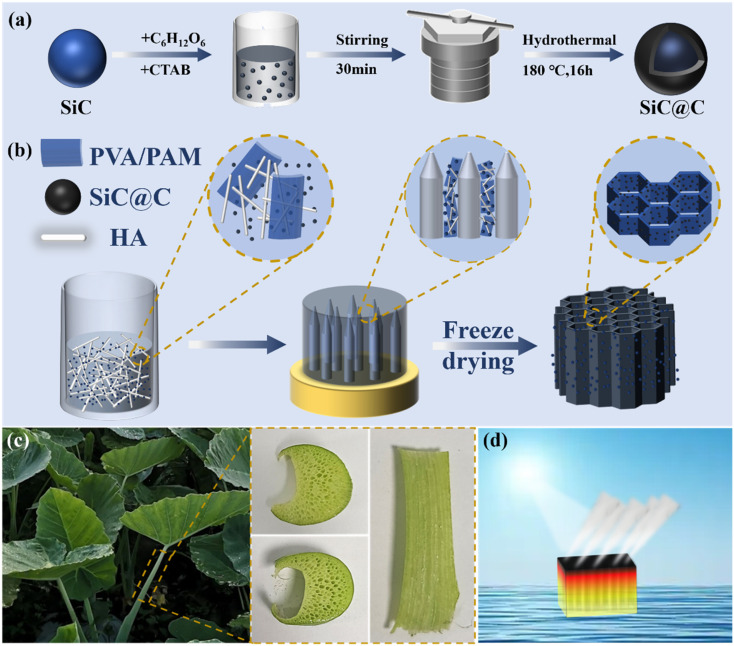
Schematic illustration: (a) the preparation of SiC@C composites with core–shell structure; (b) the preparation process of biomimetic SiC@C/HPP aerogels with vertically ordered channels; (c) digital images of the taro stem structure, including the cross-section and the side-section; (d) solar-driven water evaporation using biomimetic SiC@C/HPP aerogel.

Inspired by this, we utilized a unidirectional freezing ice templating method to mimic the porous structure found within taro stems, thereby creating vertically ordered channels within the aerogel. Fig. 1 illustrates the preparation process of the SiC@C/HPP aerogel. First, core–shell SiC@C nanoparticles were synthesized *via* a hydrothermal method using glucose as the carbon source and CTAB as the surfactant (Fig. 1a). We then mixed SiC@C nanoparticles with HA nanorods, PVA and PAM to form a composite suspension. Using a unidirectional freezing ice templating method, we prepared the biomimetic SiC@C/HPP aerogel (Fig. 1b). This process guided the longitudinal growth of ice crystals. Meanwhile, the HA/PVA/PAM mixture and SiC@C composites were squeezed into the gaps between the growing ice crystals. During subsequent freeze-drying, the ice crystals sublimated and left behind vertically aligned channels. The solute-rich regions between ice crystals, containing SiC@C and HA nanorods, became the pore walls. Because the HA nanorods and SiC@C particles are sufficiently small, they were easily pushed into these interstices by the advancing ice front. Notably, the cross-sectional structure of the SiC@C/HPP aerogel strongly resembles that of natural taro stems (Fig. 1c).

This biomimetic aerogel uses SiC@C nanoparticles as light absorbers. Hydroxyapatite (HA) nanorods serve as a thermal insulation layer to minimize heat loss during evaporation. Meanwhile, PVA and PAM form the pore wall matrix. Through strong hydrogen bonding, they create a double network structure that improves the mechanical properties of the aerogel. Owing to the vertical channels inspired by taro stems, the biomimetic SiC@C/HPP aerogel can rapidly transport water to the evaporation interface, promoting vapour generation (Fig. 1d). After condensation, the steam yields clean water. Thus, the material is suitable for seawater desalination and wastewater treatment.

**Fig. 2:**
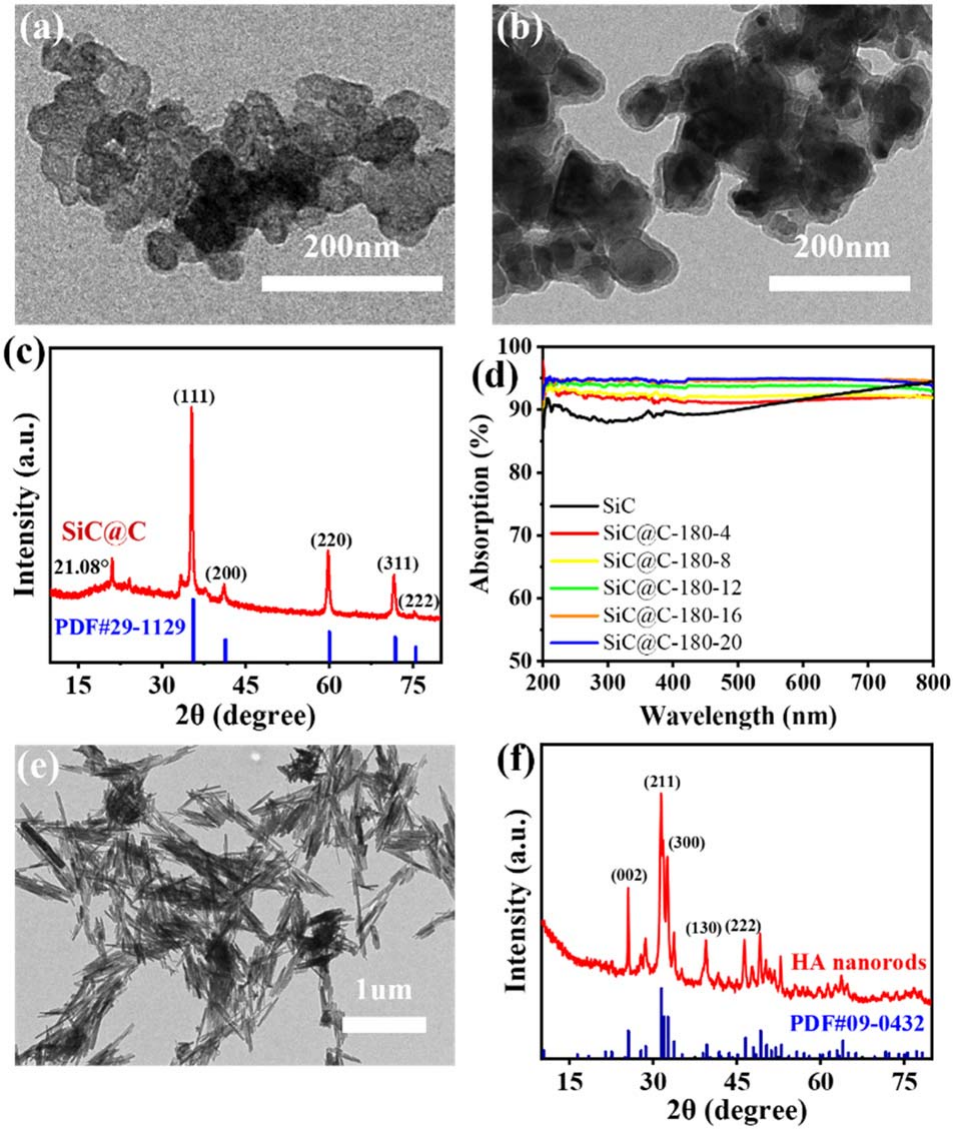
TEM, XRD and light absorption characterization of samples: (a) TEM micrographs of pure SiC; (b) TEM micrographs of SiC@C composites; (c) XRD patterns of SiC@C composites; (d) ultraviolet-visible (UV-vis) absorption spectra of SiC and SiC@C composites with different processes; (e) TEM micrographs of the HA nanorods; (f) XRD patterns of the HA nanorods.

The structure and morphology of SiC and SiC@C composites were characterized using transmission electron microscopy (TEM) and X-ray diffraction (XRD). As shown in Fig. 2a, pure SiC particles exhibited a nanoscale diameter of approximately 40 nm. TEM images of the SiC@C composite clearly revealed its core–shell structure (Fig. 2b). Furthermore, the XRD pattern confirmed the successful synthesis of the SiC@C composite (Fig. 2c). The characteristic diffraction peaks of the composite matched well with the standard PDF card for 3C–SiC, while a peak around 21.08° indicated the successful incorporation of carbon material. The thickness of the carbon layer in SiC@C composites was controlled by varying the hydrothermal conditions. As shown in Fig. S1 (SI), TEM analysis was performed on SiC@C composites prepared under different hydrothermal temperatures and durations. TEM images reveal that at 180 °C, the carbon layer thickness increased from 5 nm to 17 nm as the reaction time was extended. However, as the reaction temperature and duration were further increased, larger carbon nanospheres gradually formed within the samples. We propose that excessively high temperatures or prolonged reaction times may promote carbon self-assembly into nanospheres, rather than leading to uniform coating on the SiC substrate.

UV-vis spectroscopy was used to compare the optical properties of SiC@C and pure SiC. The composite showed substantially enhanced light absorption across the 200–800 nm range, confirming that the carbon coating improves light harvesting (Fig. 2d). The photothermal performance of the composite was then evaluated. Under 1 sun irradiation (1 kW m^−2^), the SiC@C composite reached a maximum temperature of 83 °C, significantly surpassing both pure SiC and pure carbon materials (Fig. S2a, SI). This demonstrates that the incorporation of carbon effectively enhances the photothermal conversion performance of the material. After 10 heating–cooling cycles, the material consistently maintained stable heating above 80 °C, confirming its excellent cyclic stability (Fig. S2b, SI).

The composition and morphology of the hydroxyapatite (HA) nanorods were examined by transmission electron microscopy (TEM), scanning electron microscopy (SEM) and X-ray diffraction (XRD). TEM images (Fig. 2e) and SEM images (Fig. S3, SI) reveal that the HA nanorods have a length of approximately 1 to 2 µm and a diameter of about 10 nm. The XRD patterns confirmed the successful synthesis of the HA nanorods, with their diffraction peaks matching those of the standard PDF card (Fig. 2f). These HA nanorods can serve as a thermal insulation framework for the biomimetic aerogel. They reduce heat loss from the evaporator while enhancing the mechanical properties. Moreover, the dimensions of the HA nanorods allow them to be easily displaced into the interstitial spaces between ice crystals during ice crystal growth, without interfering with the formation of vertical channels (Fig. 1b).

**Fig. 3:**
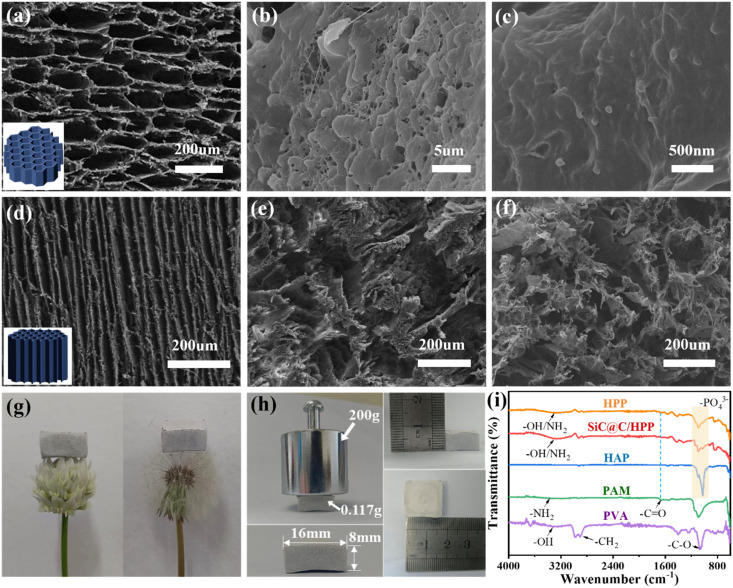
Characterization and mechanical properties of biomimetic SiC@C/HPP aerogels: (a) SEM micrograph of the cross-section; (b and c) SEM micrographs of the tube wall; (d) SEM micrograph of longitudinal-section; (e and f) cross-sectional SEM micrograph of disordered aerogel obtained *via* non directional freezing; (g) biomimetic aerogel structures on a flower (left) and a dandelion (right); (h) density, dimensional stability, and compressive resistance measurements; (i) FTIR spectra of HPP aerogel, SiC@C/HPP aerogel, HA nanorods, PVA, and PAM.

Scanning electron microscopy (SEM) was used to characterize the morphology of both the biomimetic SiC@C/HPP aerogel and a disordered aerogel. The disordered aerogel served as a control sample. It was prepared by freezing in a conventional refrigerator, while keeping all other steps unchanged. As shown in Fig. 3a–d, the cross-section of the biomimetic SiC@C/HPP aerogel reveals a honeycomb-like porous network. The pore diameter is about 80 µm, and the pore wall thickness is about 1 µm. Moreover, Fig. 3c clearly shows distinct particles and rod-like protrusions on the aerogel tube walls. This observation confirms that the tube walls consist of a composite material: SiC@C photothermal particles, hydroxyapatite (HA) nanorods, and a PVA/PAM double network structure. The biomimetic SiC@C/HPP aerogel exhibits vertically aligned, parallel channels. This structure closely resembles the internal structure of the taro stem (Fig. S4, SI). In contrast, Fig. 3e and f show the internal channels of the aerogel prepared by freezer freezing. These channels are tortuous and disordered, and the pore sizes are highly non-uniform. This is significantly different from the biomimetic aerogels with vertically ordered channels obtained *via* the unidirectional freezing ice templating method.

The vertically aligned channels within the honeycomb-like pore structure result from the ice-template effect during directional freezing. A unidirectional vertical temperature gradient drives the longitudinal growth of ice crystals during this process. The SiC@C/HA/PVA/PAM composite is simultaneously expelled into the interstitial spaces between the growing ice crystals (Fig. 1b). Subsequent freeze-drying causes the ice crystals to sublime, leaving behind the SiC@C/HA/PVA/PAM mixture that retains the channel morphology after ice removal.^54^ Consequently, the biomimetic SiC@C/HPP aerogel exhibits parallel, vertical channels that closely resemble the structure of a taro stem (Fig. 1c).

The mechanical properties of the biomimetic SiC@C/HPP aerogel were tested. As shown in Fig. 3g, the aerogel can rest on delicate flowers and dandelions without damaging their structures. Moreover, a 0.117 g sample supported a load of 200 g, equivalent to 1709 times its own weight, without noticeable deformation (Fig. 3h). The density of this biomimetic aerogel is 0.0571 g cm^−3^. These results demonstrate that the aerogel combines low density with high compressive strength.

Fourier transform infrared (FTIR) spectroscopy characterized HPP aerogels, SiC@C/HPP aerogels, HA nanorods, PVA, and PAM. Fig. 3i displays characteristic peaks: HA nanorods (OH group: 3579.7 cm^−1^; PO_4_^3−^ group: 1095.71, 1026.07, 960.40 cm^−1^), PVA (OH group: 3330.90 cm^−1^; C–O group: 1078.15 cm^−1^), PAM (NH_2_ group: 3361.76 cm^−1^; C=O group: 1664.24 cm^−1^). FTIR spectroscopy of the HPP aerogel shows characteristic peaks corresponding to HA nanorods, PVA, and PAM, confirming the successful incorporation of all three components. Notably, in the FTIR spectrum of the SiC@C/HPP aerogel, the characteristic peaks of PAM (3361.76 cm^−1^) and PVA (3330.90 cm^−1^) both shift to 3263.39 cm^−1^. In contrast, physically blending PVA and PAM caused no significant peak shift (Fig. S5, SI). Thus, in the biomimetic aerogel, hydrogen bonds between hydroxyl groups of PVA and amino groups of PAM form a double network structure.^42^

The mechanical properties of the biomimetic SiC@C/HPP aerogel and a disordered aerogel were characterized. As shown in Fig. S6a (SI), compressive stress–strain curves were compared for the PAM aerogel, PVA aerogel, PVA/PAM aerogel, HPP aerogel, SiC@C/HPP aerogel, and the disordered aerogel. At 70% compressive strain, the HPP aerogel withstood a stress of 926.7 kPa, which was significantly higher than that of the PAM aerogel (28.5 kPa), PVA aerogel (765.1 kPa), PVA/PAM aerogel (844.6 kPa), and the disordered aerogel (354.2 kPa). Similarly, tensile tests (Fig. S6b, SI) showed that the HPP aerogel reached a maximum tensile stress of 376.29 kPa, markedly exceeding the values for the PVA/PAM aerogel (344.85 kPa), PAM aerogel (20.27 kPa), and PVA aerogel (322.31 kPa). These results demonstrate that the PVA/PAM double network structure formed by strong hydrogen bonding significantly enhances the aerogel's mechanical properties. In addition, the incorporation of HA nanorods also improves the mechanical properties. This confirms their role as a thermal insulation framework. The addition of SiC@C may slightly alter the microstructure of the biomimetic aerogel. Nevertheless, under 70% compressive strain, the biomimetic SiC@C/HPP aerogel still sustained a compressive stress of 523.1 kPa, outperforming many reported aerogel materials (Fig. S7, and Table S1, SI).^57–65^

Additionally, HPP aerogels with varying HA and PVA/PAM mass ratios (1 : 1, 1 : 2, 1 : 3, 1 : 4) were examined for their morphology, mechanical properties, and water absorption capability. The internal pore diameter of natural taro stems varies considerably along their length because their thickness is not uniform (Fig. 1c). Therefore, we systematically optimized the pore structure of the aerogel by adjusting the component ratios and process parameters, and finally established the optimal preparation procedure. All samples showed vertically aligned pores with diameters ranging from 40 to 150 µm (Fig. S8, SI). The pore size gradually decreased as the PVA/PAM content increased. This trend can be attributed to the higher polymer content promoting a greater cross-linking density and thus reducing the pore dimensions. Mechanical tests revealed that the compressive strength at 70% strain increased from 241.5 kPa (1 : 1) to 724.4 kPa (1 : 4) with a higher PVA/PAM ratio (Fig. S9a and b, SI). Meanwhile, the maximum tensile stress that the HPP aerogels could withstand showed an initial increase followed by a decrease, peaking at 388.6 kPa for the 1 : 2 ratio before declining to 223.2 kPa at the 1 : 4 ratio (Fig. S9c and d, SI). The saturated water absorption capacity was also evaluated for aerogels with different mass ratios (Fig. S10, SI). The water absorption first increased and then decreased with increasing PVA/PAM content. The highest saturated absorption, 19.4 g g^−1^, was observed at a HA-to-PVA/PAM mass ratio of 1 : 3. Considering both mechanical performance and water-absorption ability, a HA-to-PVA/PAM mass ratio of 1 : 2 was selected for the preparation of the biomimetic HPP aerogel.

We further investigated the influence of different PVA and PAM mass ratios (1 : 1, 3 : 1, 5 : 1, 8 : 1, 10 : 1) on the microstructure, mechanical properties, and water absorption capacity of HPP aerogels. All HPP aerogels exhibited honeycomb-like pore channels that closely resembled the structure of taro stem. The pore size gradually decreased as the PVA mass fraction increased (Fig. S11, SI). Mechanical testing showed that the compressive strength of HPP aerogels at 70% strain first increased and then decreased, peaking at a PVA : PAM ratio of 8 : 1 with a compressive stress of 1072.6 kPa (Fig. S12a and b, SI). In contrast, the tensile strength increased monotonically with PVA content, reaching a maximum of 490.75 kPa at a ratio of 10 : 1 (Fig. S12c and d, SI). Water absorption tests on HPP aerogels with varying PVA : PAM ratios (1 : 1–10 : 1) revealed that the saturated water absorption first rose and then declined with increasing PVA proportion, peaking at 18.9 g g^−1^ at a 5 : 1 ratio (Fig. S13, SI). Considering both mechanical performance and water absorption capacity, a PVA : PAM mass ratio of 8 : 1 was selected as the optimal composition for the aerogel.

**Fig. 4:**
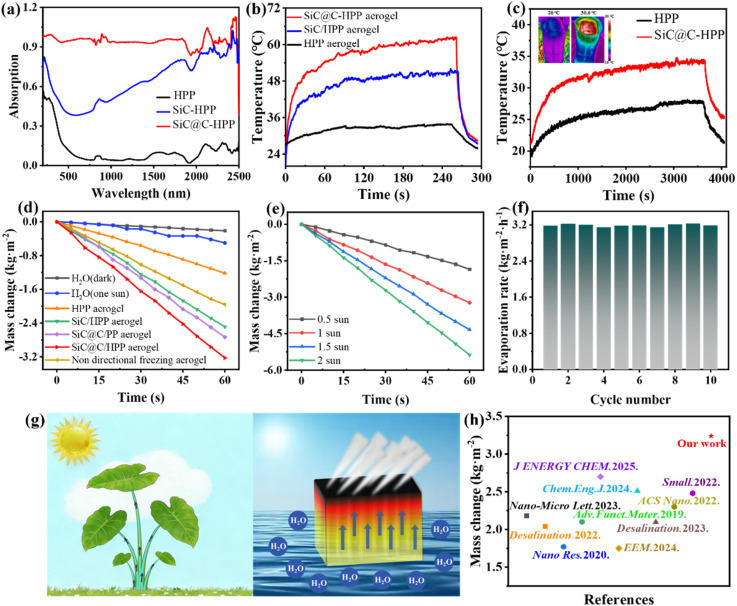
Comparative optical, photothermal, and solar evaporation performance of biomimetic SiC@C/HPP aerogels: (a) UV-vis-NIR absorption spectra of HPP, SiC/HPP, and SiC@C/HPP aerogels; (b) temperature evolution curves for dried HPP aerogel, SiC/HPP aerogel and SiC@C/HPP aerogel under 1 sun illumination(1 kW m^−2^); (c) temperature evolution curves during water evaporation for HPP and SiC@C/HPP under 1 sun illumination (1 kW m^−2^), with insets showing infrared thermal images after 10 min irradiation (left: HPP, right: SiC@C/HPP); (d) mass change of pure water under dark conditions, pure water under 1 sun illumination (1 kW m^−2^), and water in the presence of biomimetic HPP, SiC/HPP, SiC@C/PP, SiC@C/HPP, and non-directionally frozen disordered SiC@C/HPP aerogels *versus* irradiation time; (e) changes in the mass of biomimetic SiC@C/HPP aerogel as a function of irradiation time under 0.5, 1, 1.5 and 2 suns; (f) cyclic evaporation stability of SiC@C/HPP over 10 consecutive 1 sun illumination cycles; (g) schematic diagrams of transpiration in natural taro plants (left) and biomimetic aerogel applications for solar-driven water evaporation (right); (h) comparative evaporation performance benchmarked against literature reports (Table S2, SI).

UV-vis-NIR spectroscopy with integrating sphere characterized the optical properties of HPP aerogel, SiC/HPP aerogel, and SiC@C/HPP aerogel (Fig. 4a). Compared to HPP aerogel, the biomimetic SiC@C/HPP aerogel demonstrates superior broadband light absorption across the 200–2500 nm spectrum. In contrast, the absorption of SiC/HPP aerogels is mainly confined to the ultraviolet-visible region. This enhanced absorption originates from the main factor that the carbon coating significantly improves the light absorption of SiC.

The photothermal conversion performance of the biomimetic SiC@C/HPP aerogel was evaluated. Fig. S14a (SI) illustrates the indoor test setup. As shown in Fig. 4b, under 1 sun illumination (1 kW m^−2^), the surface temperature of the biomimetic SiC@C/HPP aerogel in the dry state reached up to 60.4 °C. In contrast, the HPP aerogel and SiC/HPP aerogel reached only 33.9 °C and 51.7 °C, respectively. This further demonstrates that the core–shell structured SiC@C composite has superior photothermal conversion performance, surpassing that of pure SiC. Subsequently, the influence of different solar irradiance levels (0.5, 1, 1.5, and 2 suns) on the photothermal conversion performance of the biomimetic SiC@C/HPP aerogel was investigated. As shown in Fig. S14b (SI), the surface temperature of the aerogel in the dry state increased monotonically from 53.5 °C to 81.0 °C with increasing irradiance. Furthermore, ten heating–cooling cycles were performed under 1 sun illumination (1 kW m^−2^). The results show that the aerogel consistently reached 60.0 ± 0.8 °C within 10 minutes in each cycle (Fig. S14c, SI). This demonstrates the excellent thermal and cycling stability of the biomimetic SiC@C/HPP aerogel.

The photothermal performance of the biomimetic SiC@C/HPP aerogel was tested during solar-driven water evaporation. Under 1 sun illumination (1 kW m^−2^), the surface temperature of the biomimetic SiC@C/HPP aerogel rose to 34.6 °C within 10 minutes. By comparison, under the same conditions, the surface temperature of the HPP aerogel reached only 27.8 °C (Fig. 4c). Taken together, these results demonstrate that the biomimetic SiC@C/HPP aerogel possesses exceptional photothermal conversion capability.

The effect of SiC@C loading on the photothermal performance, water absorption capacity, and evaporation properties of the biomimetic aerogel was investigated. Fig. S15 (SI) displays infrared thermal images of different biomimetic SiC@C/HPP aerogels under 1 sun illumination (1 kW m^−2^). The results show that the surface temperature of the aerogel gradually increased with higher SiC@C content (0.05, 0.08, 0.10, 0.12, and 0.15 g). Moreover, the top surface temperature was significantly higher than that of the bottom, indicating effective thermal localization. Photothermal performance tests further confirmed this trend (Fig. S16a, SI): the surface temperature of the biomimetic SiC@C/HPP aerogel rose from 43.1 °C to 68.5 °C as the SiC@C content increased. Water absorption tests revealed that the saturated water-absorption capacity first increased and then decreased with increasing SiC@C loading. The best absorption performance, 18.78 g g^−1^, was achieved at a loading of 0.08 g SiC@C (Fig. S16b, SI). This is mainly because SiC@C nanoparticles enhance the photothermal properties of the aerogel, but excessive SiC@C loading obstructs the vertical channels, thereby reducing its water-absorption capacity.

Similarly, under 1 sun illumination (1 kW m^−2^), the water evaporation rate of the aerogel first increased and then decreased with higher SiC@C content. At SiC@C loadings of 0.05, 0.08, 0.10, 0.12, and 0.15 g, the evaporation rates were 2.31, 2.88, 3.24, 3.07, and 2.76 kg m^−2^ h^−1^, respectively (Fig. S17, SI). Therefore, considering the overall photothermal efficiency, water absorption capacity, and evaporation rate, a SiC@C loading of 0.10 g was selected as the optimum.

The influence of aerogel thickness on evaporation performance was investigated. When the biomimetic SiC@C/HPP aerogel was 7, 10, 12, and 15 mm thick, the corresponding water evaporation rates were 2.92, 3.19, 3.00, and 2.71 kg m^−2^ h^−1^, respectively (Fig. S18, SI). This trend of first increasing and then decreasing results from two competing mechanisms: (1) an excessively thin aerogel allows partial heat dissipation to the underlying water, leading to thermal loss; (2) an overly thick aerogel impedes capillary-driven water transport, thereby reducing the evaporation rate. Therefore, 10 mm was selected as the optimal thickness for the biomimetic SiC@C/HPP aerogel.

The solar evaporation performance of the biomimetic SiC@C/HPP aerogel was evaluated against several control samples. As shown in Fig. 4d, the mass loss of pure water in the dark was only 0.10 kg m^−2^ after one hour. Under 1 sun illumination (1 kW m^−2^), the mass loss rates for pure water, the HPP aerogel, the SiC/HPP aerogel, the SiC@C/PP aerogel (without HA nanorods), the SiC@C/HPP aerogel, and the disordered aerogel reached 0.50, 1.22, 2.49, 2.73, 3.24, and 1.96 kg m^−2^ h^−1^, respectively. The results show that the water evaporation rate of the biomimetic SiC@C/HPP aerogel significantly exceeds that of the HPP and SiC/HPP aerogels. This improvement is attributed to the carbon coating, which greatly enhances the light absorption of silicon carbide and thereby increases photothermal conversion efficiency. Moreover, the evaporation performance of the SiC@C/PP aerogel (lacking HA nanorods) was markedly lower than that of the SiC@C/HPP aerogel. This demonstrated the thermal insulation effect of the HA nanorods in reducing heat loss. Most notably, the biomimetic SiC@C/HPP aerogel with vertically aligned channels outperformed the disordered aerogel in evaporation performance, highlighting the critical role of the biomimetic structure in enabling rapid water transport and enhancing overall evaporation efficiency.

The water evaporation performance of the biomimetic SiC@C/HPP aerogel was tested under different solar irradiance levels. As shown in Fig. 4e, the evaporation rates were 1.85, 3.24, 4.33, and 5.38 kg m^−2^ h^−1^ at irradiance levels of 0.5, 1, 1.5, and 2 suns, respectively. This indicates that the evaporation performance improves with increasing solar irradiance. Furthermore, Fig. 4f shows that the biomimetic SiC@C/HPP aerogel maintained a stable evaporation rate (approximately 3.20 kg m^−2^ h^−1^) over ten consecutive solar radiation cycles at 1 kW m^−2^. This confirms that the biomimetic evaporator possesses stable evaporation performance. To further verify its long-term stability, we performed water evaporation tests for ten hours per day over three consecutive days (Fig. S19, SI). The results show that during the 30-hour test, the evaporation rate of the biomimetic SiC@C/HPP aerogel remained stable without significant fluctuations.

The solar-driven water evaporation mechanism of the biomimetic aerogel structure was investigated. As shown in Fig. 4g, the natural taro plant is a typical aquatic plant. Its petiole (taro stem) has evolved into an efficient transport structure that can rapidly convey water and nutrients to the apical leaves. The interior of the taro stem contains a neatly arranged vascular bundle structure.^56^ The vessel walls have a loose tissue arrangement, forming numerous longitudinally penetrating channels (Fig. 1c). These channels facilitate gas circulation and water transport. Inspired by this natural architecture, we used a unidirectional freezing ice templating method to construct vertical biomimetic channels within the aerogel (Fig. 1b). This vertical channel structure closely resembles the porous structure found inside the taro stem. As a result, the biomimetic aerogel can quickly transport water from the base to the upper evaporation interface, thereby improving the evaporator's performance. Calculations indicate that the total production cost of this biomimetic evaporator is approximately $25.59 m^−2^. This includes raw material costs of 15.72 m^−2^ and additional expenses for liquid nitrogen and energy consumption amounting to $9.87 m^−2^. Compared with evaporators reported in other studies, the biomimetic SiC@C/HPP aerogel outperforms most solar-driven evaporators (Fig. 4h, and Table S2, SI).^53,66–74^ Therefore, although the cost is slightly higher than that of some evaporators, the evaporator described in this study shows superior evaporation performance. Moreover, while liquid nitrogen-assisted unidirectional freezing technology still requires further development for industrial-scale application, most of the materials used to construct the evaporator are already produced at large scale. This suggests strong potential for commercial application (Table S3, SI).^75–79^ This outstanding performance arises from three synergistic mechanisms: rapid capillary-driven water transport enabled by the biomimetic vertically aligned channels; efficient broadband light harvesting and excellent stability provided by the SiC@C material; and effective reduction of heat loss due to the insulating framework formed by the HA nanorods.

**Fig. 5:**
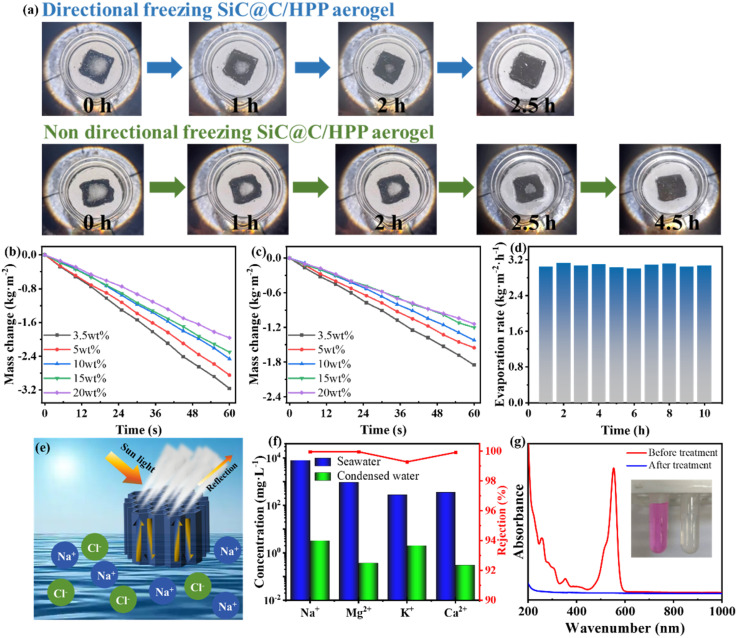
Solar desalination and wastewater treatment performance of biomimetic SiC@C/HPP aerogels. (a) Salt resistance test: photographs showing dissolution of surface-deposited NaCl (300 mg) on directionally frozen (biomimetic) and non-directionally frozen (disordered) aerogels under 1 sun illumination (1 kW m^−2^); (b) water evaporation rates of biomimetic SiC@C/HPP aerogels in NaCl solutions of different concentrations (3.5, 5, 10, 15 and 20 wt%); (c) water evaporation rates of disordered aerogels in NaCl solutions of different concentrations (3.5, 5, 10, 15 and 20 wt%); (d) continuous water evaporation rate of the biomimetic SiC@C/HPP aerogel in natural Bohai Sea seawater (40.6°N, 120.8°E) over 10 hours; (e) mechanistic illustration of salt rejection capability through vertically aligned microchannels; (f) concentrations and corresponding ion rejection rates of the four major ions (Na^+^, Mg^2+^, K^+^, Ca^2+^) in the natural seawater and the collected condensate; (g) UV-vis absorption spectra of the simulated wastewater containing rhodamine B (10 mg L^−1^) and the condensate after evaporation, with corresponding photographs shown in the inset.

The desalination and wastewater purification capabilities of the biomimetic SiC@C/HPP aerogel were evaluated using natural seawater and simulated wastewater. For the salt-tolerance test, 300 mg of solid NaCl particles were placed on the surfaces of the biomimetic SiC@C/HPP aerogel and the disordered aerogel (Fig. 5a). Under 1 sun illumination (1 kW m^−2^), the salts on the biomimetic SiC@C/HPP aerogel dissolved completely within 2.5 hours. In contrast, the disordered aerogel required 4.5 hours, and its dissolution rate gradually slowed. This demonstrates that the vertical channels in the biomimetic structure accelerate water transport, thereby facilitating salt diffusion. Furthermore, the salt tolerance of the biomimetic SiC@C/HPP aerogel was evaluated in NaCl solutions of varying concentrations. Under 1 sun illumination, the evaporation rate reached 3.16 kg m^−2^ h^−1^ in a 3.5 wt% NaCl solution. Even under high-salinity conditions (20 wt%), the evaporation rate remained at 1.96 kg m^−2^ h^−1^ (Fig. 5b). In contrast, the evaporation rate of the disordered aerogel decreased from 1.85 kg m^−2^ h^−1^ to 1.14 kg m^−2^ h^−1^ as the NaCl concentration increased from 3.5 wt% to 20 wt% (Fig. 5c). Although the pore size distribution and porosity of disordered aerogels differ from those of bio-inspired ones, their performance differs markedly. Therefore, the superior performance of the bio-inspired aerogel can be mainly attributed to its vertical pore structure. This further demonstrates the stable and high evaporation performance of the biomimetic aerogel under high-salinity conditions.

The seawater desalination capability of the biomimetic SiC@C/HPP aerogel was validated using natural seawater collected from the Bohai Sea (40.6°N, 120.8°E). Under 1 sun illumination (1 kW m^−2^), the evaporation rate of the aerogel remained stable at approximately 3.00 kg m^−2^ h^−1^ over ten consecutive cycles in natural seawater (Fig. 5d). This demonstrated its stable and efficient photothermal evaporation performance. Moreover, after operating continuously for 10 hours in a 10 wt% NaCl solution, no visible salt deposition was observed on the surface of the biomimetic SiC@C/HPP aerogel (Fig. S20, SI). The experimental results described above demonstrate that the bio-inspired SiC@C/HPP aerogel maintains stable and highly efficient water evaporation performance. This holds true both in high-salinity environments and in natural seawater. Furthermore, this evaporator exhibits excellent salt resistance.

The mechanism of seawater desalination using the biomimetic SiC@C/HPP aerogel was investigated. As shown in Fig. 5e, inspired by the porous structure inside taro stems, we prepared a biomimetic aerogel with vertically aligned, parallel channels. These biomimetic channels efficiently transport water to the evaporation surface. At the same time, salts in seawater (such as Na^+^ and Cl^−^) diffuse rapidly along with the water flow. Because water is continuously and quickly replenished, the concentration of salt ions at the evaporation interface is effectively diluted. This prevents salt crystallization caused by local supersaturation. As a result, the operational stability and service life of the solar-driven water evaporation device are significantly enhanced.

To further evaluate the desalination performance of the biomimetic SiC@C/HPP aerogel, we compared the natural seawater with the condensed water collected after evaporation. As shown in Fig. 5f, the concentrations of four major ions (Na^+^, Mg^2+^, K^+^, Ca^2+^) were measured in both Bohai Sea seawater and the condensate. After evaporation through the biomimetic SiC@C/HPP aerogel, the concentrations of all four ions in the condensate decreased by 2–3 orders of magnitude, corresponding to ion rejection rates exceeding 99%. Notably, the ion concentrations in the condensate fell below the drinking-water standards set by the World Health Organization.^80^ Furthermore, electrical resistivity tests were performed on the natural seawater and the condensate. As shown in Fig. S21 (SI), the resistivity of the condensate reached 1906 kΩ, which is 60.7 times that of the natural Bohai Sea seawater (31.4 kΩ). Consequently, for large-scale desalination applications, the biomimetic SiC@C/HPP aerogel investigated in this study exhibits long-term stable evaporation performance and excellent ion retention capacity. These features demonstrate its strong potential for practical deployment.

Additionally, outdoor evaporation testing was conducted on the biomimetic SiC@C/HPP aerogel (Fig. S22, SI). During 9 hours of natural solar exposure (09:00–18:00), the collected condensate yield reached 5.85 kg m^−2^. This indicates that the freshwater, produced by a 1 m^2^ sample in a single day, can meet the daily drinking-water requirements of three adults (assuming 1.5–2.0 kg per person).

The wastewater treatment capability of the biomimetic SiC@C/HPP aerogel was evaluated using simulated wastewater. As shown in Fig. 5g, rhodamine B (10 mg L^−1^) was nearly completely eliminated in the condensate collected after evaporation. UV-vis absorption spectroscopy further confirmed the effective removal of rhodamine B, as its characteristic absorption peak almost disappeared. In addition, simulated wastewater containing Cr^3+^, Mn^2+^, Ni^2+^, and Pb^2+^ (each at 100 mg L^−1^) was tested. Fig. S23 (SI) shows that the concentrations of all four heavy-metal ions in the condensate collected after evaporation through the biomimetic SiC@C/HPP aerogel were below 0.15 mg L^−1^, corresponding to removal rates exceeding 99.8%. Furthermore, all components of the evaporator (SiC@C, HA nanorods, PVA, and PAM) have minimal environmental impact.^81–85^ This suggests that the biomimetic SiC@C/HPP aerogel could serve as an efficient solar-driven evaporator for seawater desalination and wastewater treatment. Its vertically ordered structure enhances water transport and salt tolerance during solar evaporation, offering a promising approach to address global freshwater scarcity.

## 4 Conclusions

Inspired by the porous structure of taro stems, we fabricated biomimetic SiC@C/HPP aerogels with vertically ordered channels using a unidirectional freezing ice templating method. In this architecture, core–shell SiC@C functions as the light absorber, hydroxyapatite (HA) nanorods serve as the thermal insulation material, and a PVA/PAM composite forms the tube wall substrate. Under 1 sun illumination (1 kW m^−2^), the evaporation rate of the biomimetic aerogel reached 3.24 kg m^−2^ h^−1^, which is significantly higher than that of the disordered structure (1.96 kg m^−2^ h^−1^). When tested in natural seawater, the evaporation rate remained at 3.00 kg m^−2^ h^−1^. The rejection rates for major seawater ions (Na^+^, Mg^2+^, K^+^ and Ca^2+^) exceeded 99%, and the ion concentrations in the condensed water were well below the World Health Organization (WHO) drinking water standards. These results demonstrate excellent seawater desalination performance. Overall, this study presents a biomimetic structural design strategy with exceptional salt tolerance, enabling high-performance solar evaporation in both seawater and brine. This approach offers a promising route for sustainable freshwater production.

## Author contributions

Zhuo Wang: writing-original draft, validation, methodology, formal analysis, data curation, conceptualization. Mengya Yu: writing-review & editing, visualization, investigation. Jiong Kong: investigation, data curation. Mi Zheng: investigation, data curation. Weifeng Li: writing-review & editing, validation, methodology, conceptualization. Yumei Long: writing-review & editing, supervision, funding acquisition, conceptualization. Zuoshan Wang: writing-review & editing, validation, supervision.

## Conflicts of interest

The authors declare that they have no known competing financial interests or personal relationships that could have appeared to influence the work reported in this paper.

## Supplementary Material

RA-016-D6RA03111K-s001

## Data Availability

The data that support the findings of this study are available from the corresponding author upon reasonable request. Supplementary information (SI): calculation of heat loss; TEM and photothermal performance testing of SiC@C composites; TEM testing of HA nanorods; SEM testing of the natural taro stem; SEM, mechanical, photothermal, water evaporation and outdoor performance testing of biomimetic aerogels; comparison table of mechanical properties; comparison table of water evaporation performance; comparison table of evaporator costs. See DOI: https://doi.org/10.1039/d6ra03111k.
