# Supplementary material for: Taro stem-inspired aerogel with vertically ordered channels for high-efficiency solar seawater desalination
Source: RSC Adv. 2026 May 12;16(27):25081–93. doi: 10.1039/d6ra03111k (PMC13163679; doi:10.1039/d6ra03111k)
Supplement: RA-016-D6RA03111K-s001 [file RA-016-D6RA03111K-s001.pdf]

Supplementary Information (SI) for RSC Advances.
This journal is © The Royal Society of Chemistry 2026

Section SI-1. Calculation of photothermal conversion efficiency

The photothermal material was sandwiched between two identically sized square quartz glass plates and peripherally secured with transparent tape. The assembly was irradiated using an 808-nm laser (Zhuhai Tengxing Optoelectronics Co., Ltd.) at a power density of 0.1 $W \cdot cm^{-2}$ for 5 minutes, followed by natural cooling to ambient temperature. Surface temperature profiles were monitored in real-time using an infrared (IR) camera (FOTRIC 246M, CEAULIGHT) interfaced with a computer, capturing both heating and cooling dynamics. The photothermal conversion efficiency (η) is as followed:

The system's total energy balance equation:

$$\Sigma_i m_i C_{pi} \frac{dT}{dt} = Q_s - Q_{loss} \tag{S1}$$

where m_i denotes the mass of the photothermal material (0.01 g) and C_{pi} its specific heat capacity. Given that the photothermal material mass is negligible relative to the quartz glass substrate, the specific heat capacity of quartz glass (0.772 $J \cdot g^{-1} \cdot {}^{\circ}C^{-1}$) was adopted for calculations. Q_S represents the energy input from 808-nm laser irradiation, while Q_{loss} accounts for heat dissipation to the surroundings. The system reached thermal equilibrium when the sample temperature attained a steady-state maximum during heating, expressed as:

$$Q_s = Q_{loss} = hS\Delta T_{max} \tag{S2}$$

where h denotes the heat transfer coefficient, S the system surface area, and ΔT_{max} the maximum temperature change. The photothermal conversion efficiency (η) is defined by:

$$\eta = \frac{hS\Delta T_{max}}{I(1-10^{-A808})} \tag{S3}$$

where I is the incident laser power density (0.1 $W \cdot cm^{-2}$) and A808 the sample absorbance at 808 nm. Within this expression, hS constitutes the sole undetermined parameter. To resolve hS, a dimensionless driving temperature θ is introduced:

$$\theta = \frac{T - T_{surr}}{T_{max} - T_{surr}} \tag{S4}$$

here, T represents the sample temperature, T_{max} its maximum attained value, and T_{surr} the ambient temperature, with τ_s denoting the system's thermal time constant.

Upon cessation of laser irradiation ($Q_s = 0$), the energy balance simplifies to:

$$\tau_s = \frac{\Sigma_i m_i C_{pi}}{hS} \tag{S5}$$

$$\frac{d\theta}{dt} = -\frac{\theta}{\tau_s} \quad , \quad t = -\tau_s \ln\theta \tag{S6}$$

Consequently, hS is derivable from the negative slope of the linear correlation between cooling time and lnθ.

Under 808-nm laser irradiation at 0.1 W·cm^{-2}, the SiC@C composite exhibited a maximum temperature change (ΔT_{max}) of 4.6 °C with an absorbance (A808) of 1.24, yielding a photothermal conversion efficiency (η) of 92.20%. In parallel, pristine SiC displayed a Δ ΔT_{max} of 4.5 °C and A808 of 0.924, corresponding to η = 87.63%. This performance differential is primarily attributed to the enhanced light-trapping capability enabled by the carbon coating layer.

Section SI-2. Heat loss calculation

The heat loss of evaporator comprises three primary mechanisms: heat radiation, heat convection, and heat conduction. These contributions are quantified as follows:

(1) Heat radiation

The value of heat radiation is governed by the Stefan-Boltzmann law:

$$Q_{rad} = \varepsilon\sigma\,(T^4_{aerogel} - T^4_{env}) \tag{S7}$$

where ε denotes the surface emissivity (≈1), σ the Stefan-Boltzmann constant (5.67×10^{-8} $W \cdot m^{-2} \cdot K^{-4}$), and $T_{aerogel}$ and T_{env} represent the surface temperature of the SiC@C/HPP aerogel and ambient temperature, respectively. Under 1 sun illumination (1 $kW \cdot m^{-2}$), $T_{aerogel}$ and T_{env} were measured at 34.6°C and 28.6°C, yielding Q_{rad} = 38.52 $W \cdot m^{-2}$.

The fractional radiative loss (η_{rad}) is subsequently derived from:

$$\eta_{rad} = \frac{Q_{rad}}{Q_{in}} \tag{S8}$$

where Q_{in} is the incident thermal energy. At one sun condition, η_{rad} is calculated as 3.85%.

(2) Heat convection

Convective heat loss is quantified via Newton's law of cooling:

$$Q_{conv} = h(T_{aerogel} - T_{env}) \tag{S9}$$

where Q_{conv} represents the convective heat flux and h the convective heat transfer coefficient (5 $W \cdot m^{-2} \cdot K^{-1}$ under natural convection conditions). Substituting the measured temperature differential ($\Delta T = T_{aerogel} - T_{env}$ = 6.0 K) yields Q_{conv} = 30.0 $W \cdot m^{-2}$.

The fractional convective loss (η_{conv}) is then determined by:

$$\eta_{conv} = \frac{Q_{conv}}{Q_{in}} \tag{S10}$$

At 1 sun illumination (1 $kW \cdot m^{-2}$), η_{conv} = 3.0%.

(3) Heat conduction

Conductive heat flux is governed by Fourier's law:

$$Q_{cond} = Cm\Delta T/At \quad (S11)$$

where Q_{cond} denotes the conductive heat flux, Cthe specific heat capacity of water (4.2 $J\cdot g^{-1}\cdot{}^{\circ}C^{-1}$), m the water mass (20 g), ΔT the temperature increment (1°C), A the evaporative surface area of the SiC@C/HPP aerogel, and t the illumination duration (3600 s). This yields a bulk heat accumulation of 55.56 $W\cdot m^{-2}$.

The fractional conductive loss (η_{cond}) is then evaluated by:

$$\eta_{cond} = \frac{Q_{cond}}{Q_{in}} \quad (S12)$$

At 1 sun illumination (1 $kW\cdot m^{-2}$), η_{cond} = 5.56%.

The total energy distribution comprises: evaporative conversion (93.07%), heat radiation (3.85%), heat convection (3.00%), and heat conduction (5.56%). The observed summation marginally exceeds theoretical unity, possibly due to experimental or computational errors.

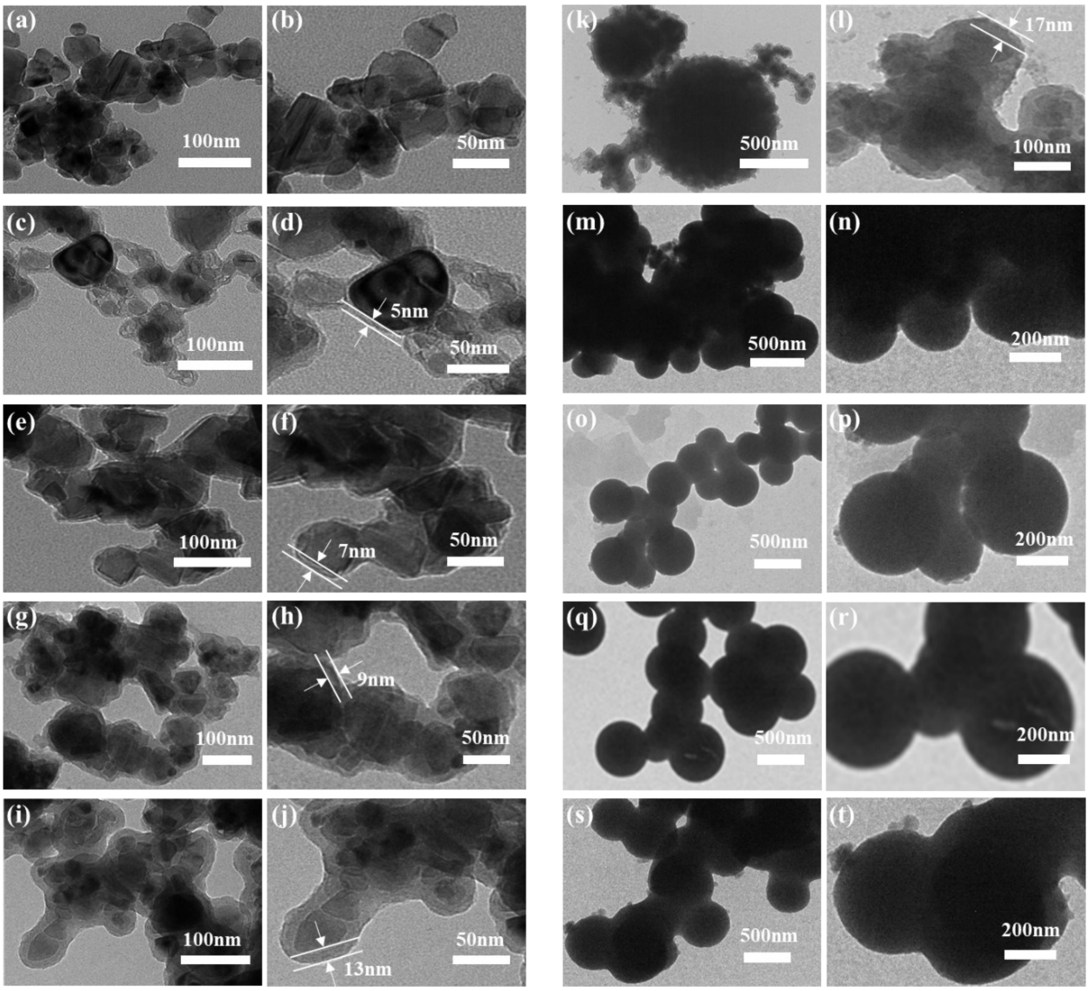

Figure S1. TEM micrographs of SiC@C composites synthesized under varied hydrothermal conditions: (a,b) 180 °C for 2 h; (c,d) 180 °C for 4 h; (e,f) 180 °C for 8 h; (g,h) 180 °C for 12 h; (i,j) 180 °C for 16 h; (k,l) 180 °C for 20 h; (m,n) 180 °C for 24 h; (o,p) 200 °C for 12 h; (q,r) 200 °C for 16 h; (s,t) 200 °C for 20 h.

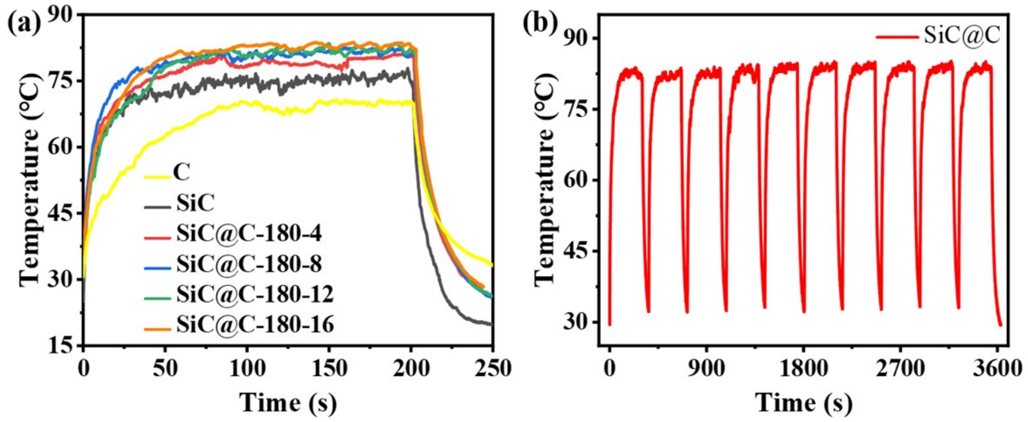

Figure S2. Photothermal performance comparisons: (a) Temperature profiles of SiC@C composites versus pristine carbon, silicon carbide, and variants synthesized under distinct hydrothermal conditions under 1 sun illumination (1 kW·m^{-2}); (b) Thermal stability assessment of SiC@C composites through 10 consecutive heating-cooling cycles.

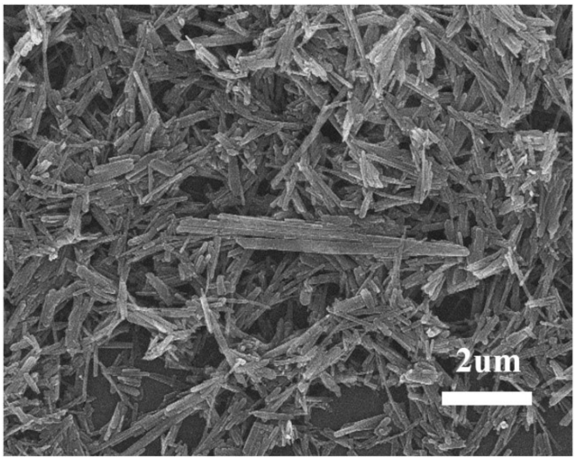

Figure S3. Transmission electron microscopy (TEM) characterization of hydroxyapatite (HA) nanorods.

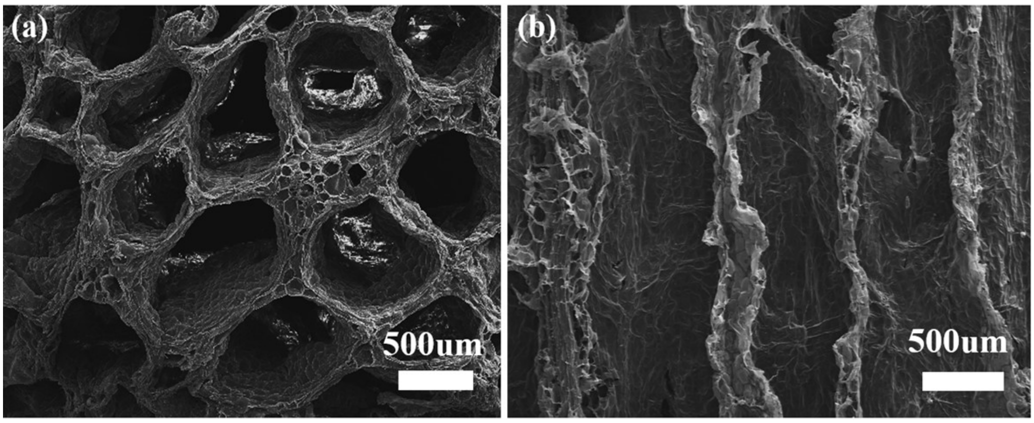

Figure S4. (a) Cross-sectional SEM image of a natural taro stem. (b) Longitudinal-section SEM image of a natural taro stem.

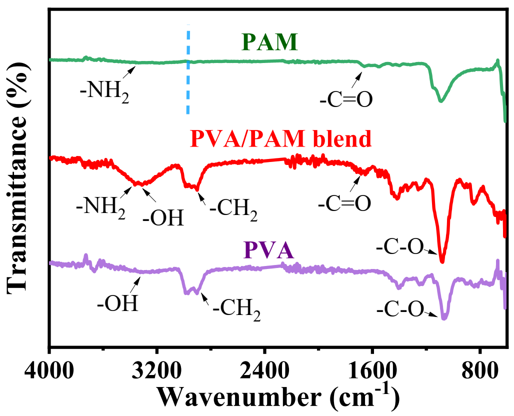

Figure S5. FTIR spectra of PAM, PVA and PVA/PAM physical blend.

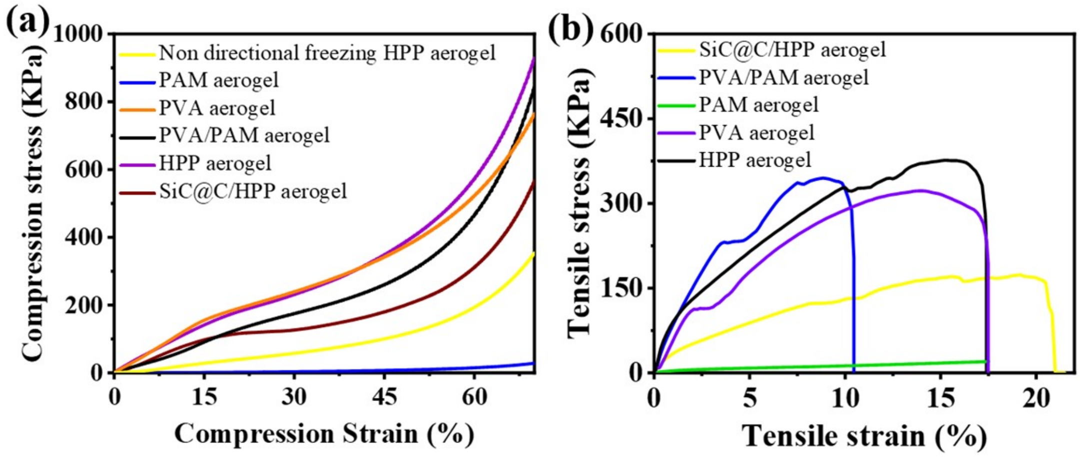

Figure S6. Mechanical property testing: (a) Compressive behavior of PAM aerogel, PVA aerogel, PVA/PAM aerogel, HA/PVA/PAM (HPP) aerogel, SiC@C/HPP aerogel and non-directionally freezing HPP aerogel under 70% compressive strain; (b) Tensile stress-strain curves of PAM aerogel, PVA aerogel, PVA/PAM aerogel, HPP aerogel and SiC@C/HPP aerogel.

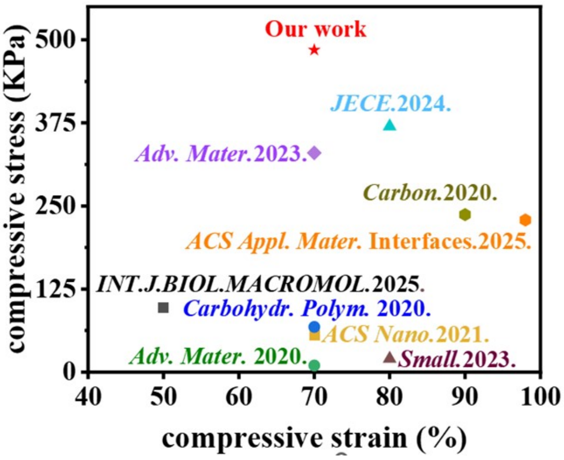

Figure S7. Comparison of the mechanical properties of biomimetic SiC@C/HPP aerogels with those of aerogels from other studies.

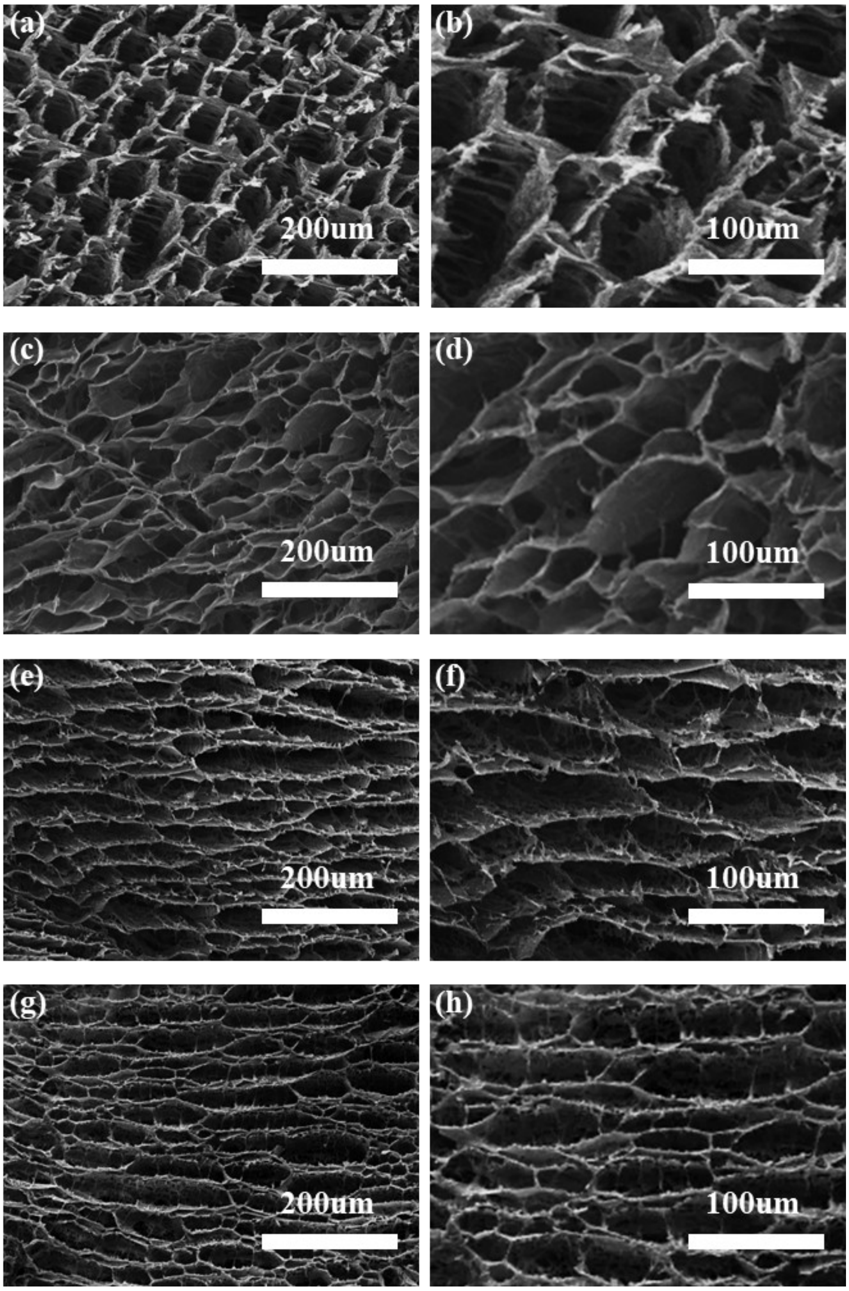

Figure S8. Cross-sectional scanning electron microscopy (SEM) analysis of biomimetic HPP aerogels with varying HA-to-PVA/PAM mass ratios: (a,b) 1:1; (c,d) 1:2; (e,f) 1:3; (g,h) 1:4.

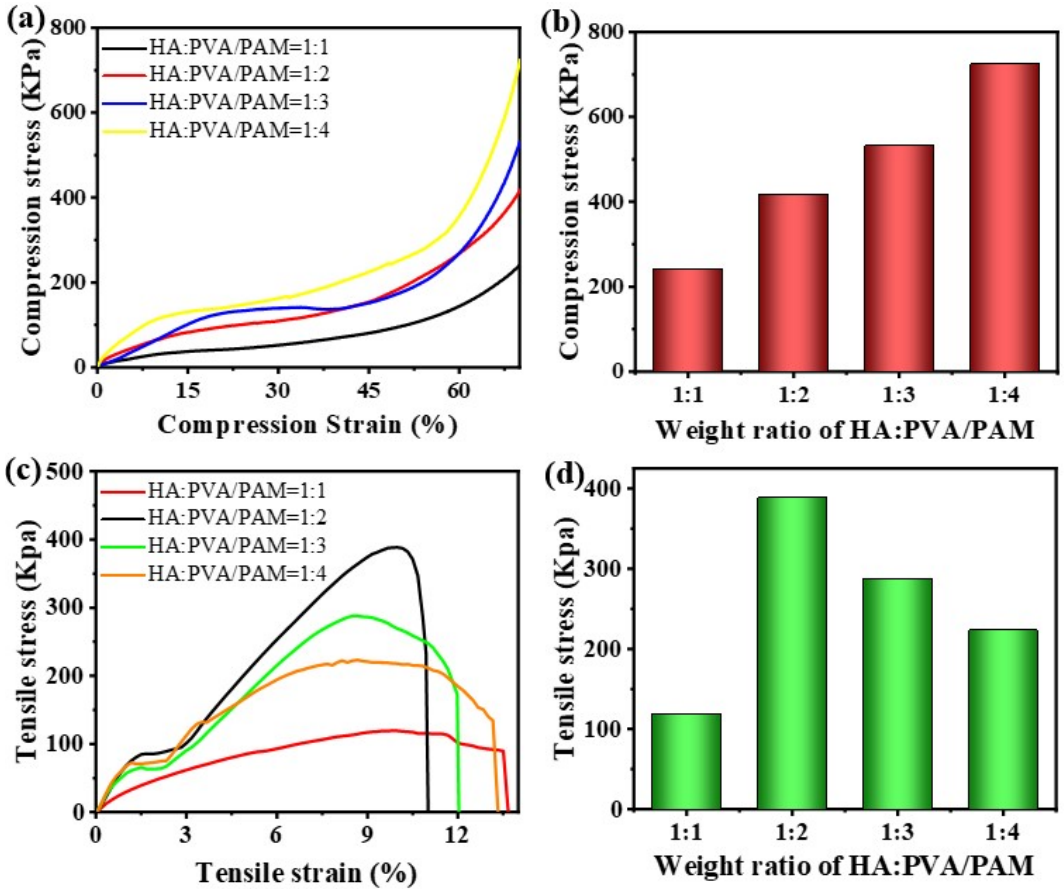

Figure S9. Mechanical performance of HPP aerogels with different HA-to-PVA/PAM mass ratios: (a) Compressive stress-strain responses; (b) Compressive strength at 70% strain; (c) Tensile stress-strain behavior; (d) Ultimate tensile strength.

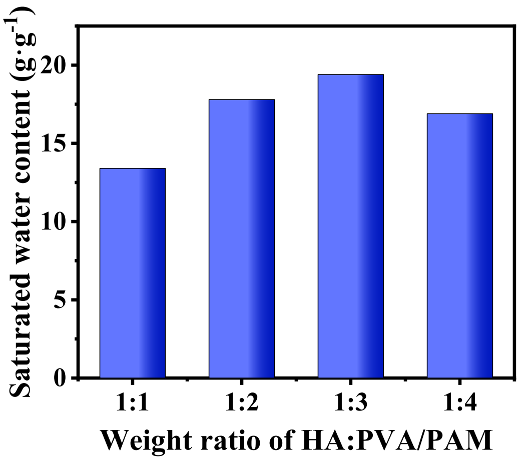

Figure S10. Equilibrium water absorption capacity of biomimetic HPP aerogels with different HAP to PVA/PAM mass ratios (1:1, 1:2, 1:3 and 1:4) after 24 h immersion.

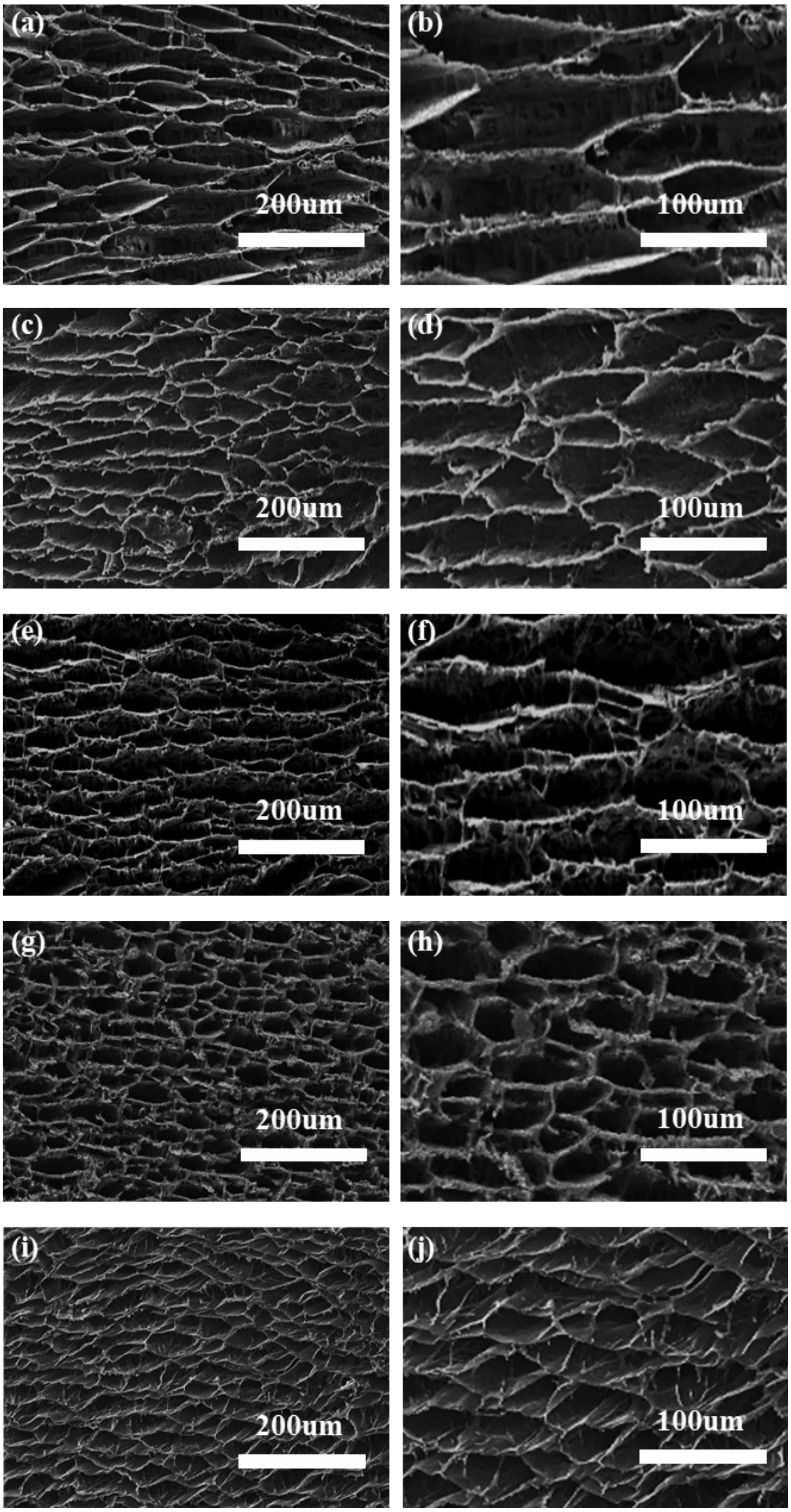

Figure S11. Cross-sectional scanning electron microscopy (SEM) analysis of biomimetic HPP aerogels with varying PVA/PAM mass ratios: (a,b) 1:1; (c,d) 3:1; (e,f) 5:1; (g,h) 8:1; (i,j) 10:1.

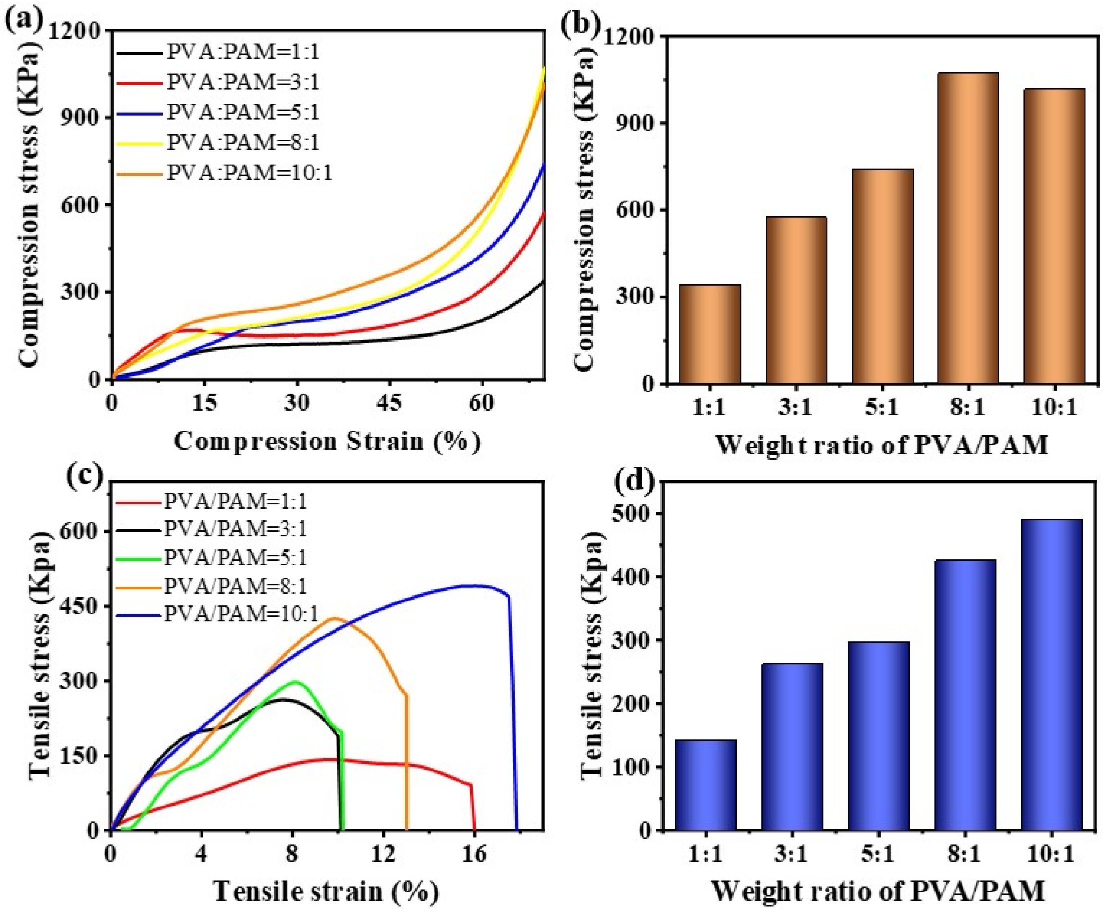

Figure S12. Mechanical performance quantification of HPP aerogels with different PVA/PAM ratios: (a) Compressive stress-strain curves; (b) Compressive strength at 70% strain; (c) Tensile stress-strain behavior; (d) Ultimate tensile strength.

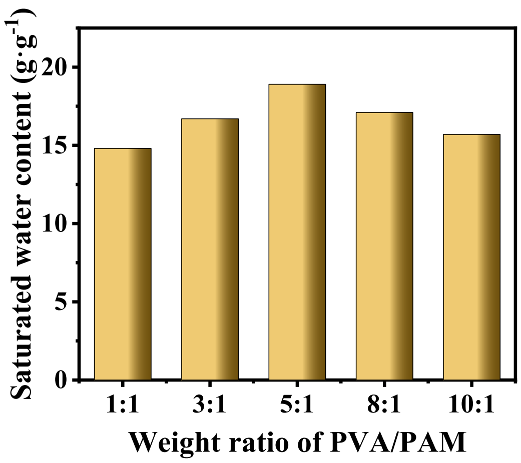

Figure S13. Equilibrium water absorption capacity of biomimetic HPP aerogels across PVA/PAM mass ratios (1:1 to 10:1), measured after 24-hour immersion.

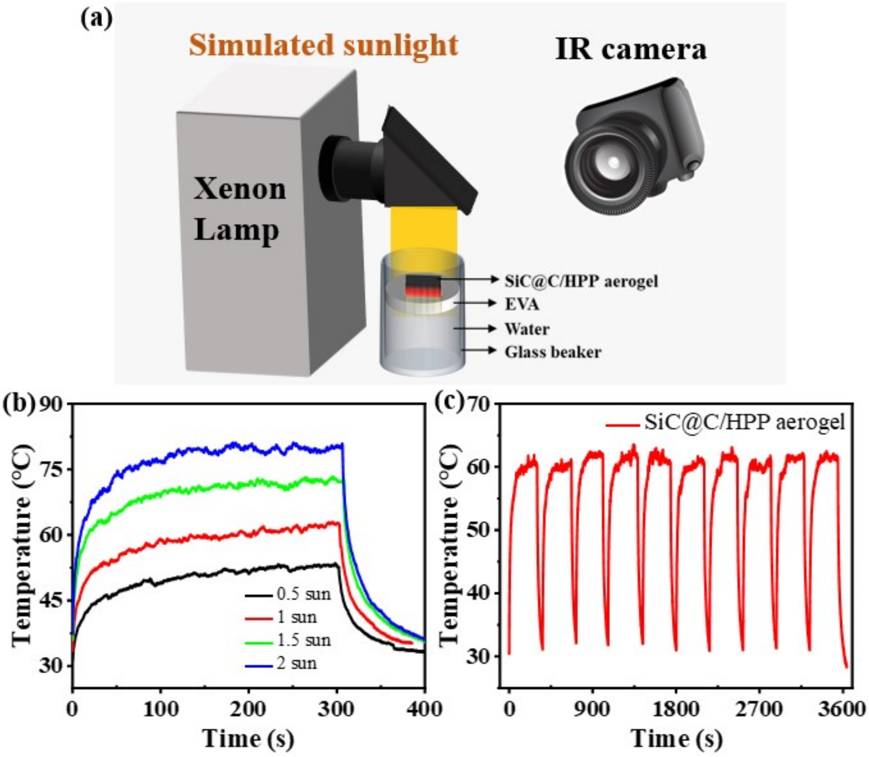

Figure S14. Photothermal characterization: (a) Experimental schematic showing solar simulation using a xenon lamp with synchronized infrared thermography; (b) Steady-state surface temperatures of SiC@C/HPP aerogel under varied solar intensities (0.5-2.0 $kW \cdot m^{-2}$); (c) Thermal cycling stability over 10 consecutive on/off cycles at 1 sun illumination.

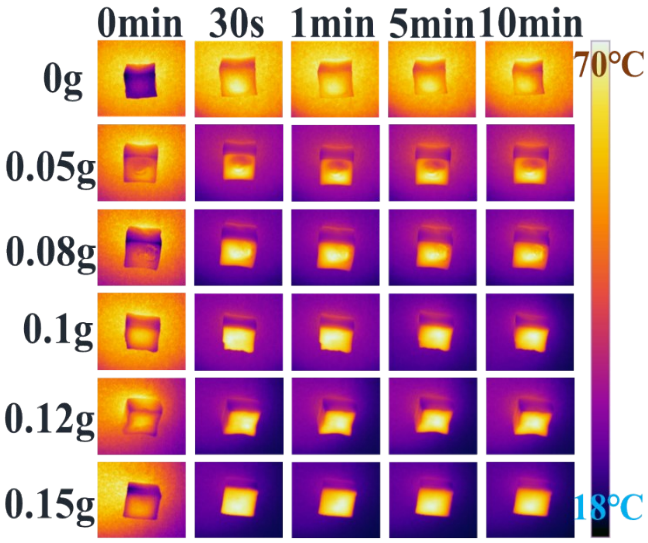

Figure S15. Infrared thermography of SiC@C/HPP aerogels with varying SiC@C mass loadings under 1 sun illumination (1 kW·m^{-2}), demonstrating surface temperature distribution during steady-state evaporation.

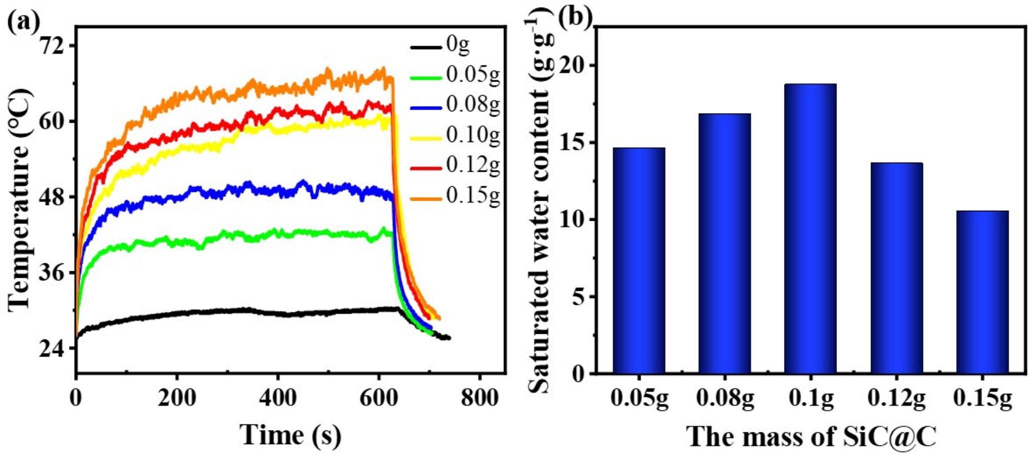

Figure S16. (a) Temporal temperature evolution profiles of aerogels with incremental SiC@C content (0, 0.05, 0.08, 0.10, 0.12, and 0.15 g) under 1 sun illumination (1 kW·m^{-2}); (b) Equilibrium water absorption capacity versus SiC@C loading after 24 h immersion.

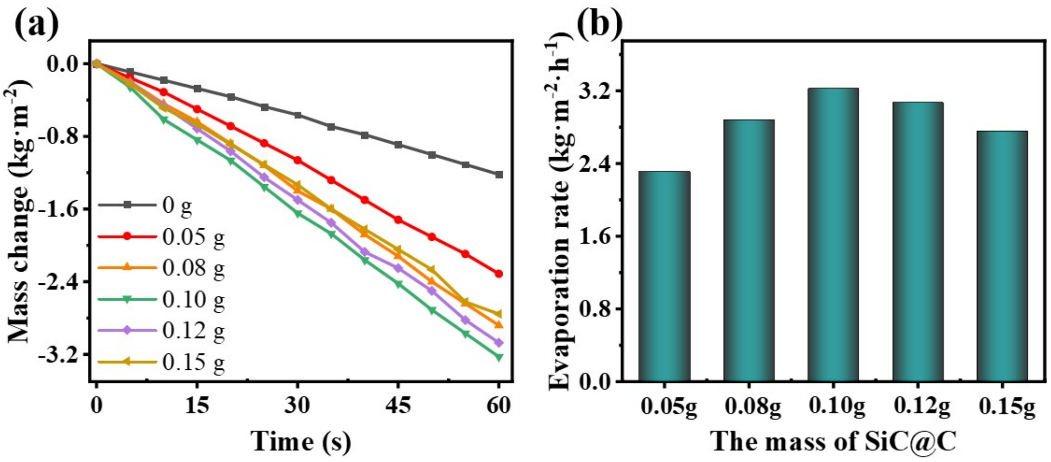

Figure S17. (a) Evaporation rates of SiC@C/HPP aerogels with varied SiC@C mass fractions; (b) Bar chart comparing the water evaporation rates of bio-inspired aerogels with different SiC@C contents.

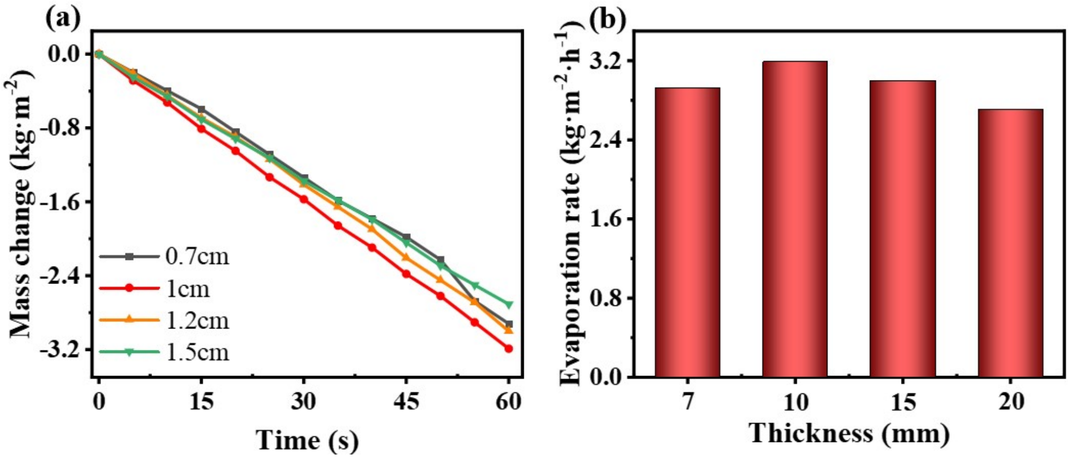

Figure S18. (a) Evaporation rate kinetics of SiC@C/HPP aerogels at thicknesses ranging from 7 to 20 mm under continuous 1 sun illumination; (b) Comparative analysis of thickness-dependent evaporation performance.

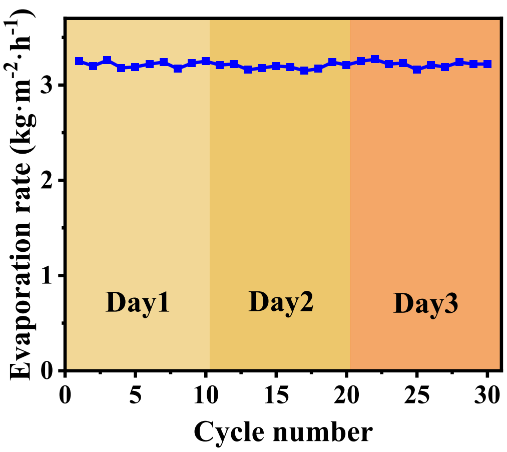

Figure S19. Cyclic evaporation stability of SiC@C/HPP over 30 consecutive 1 sun illumination cycles.

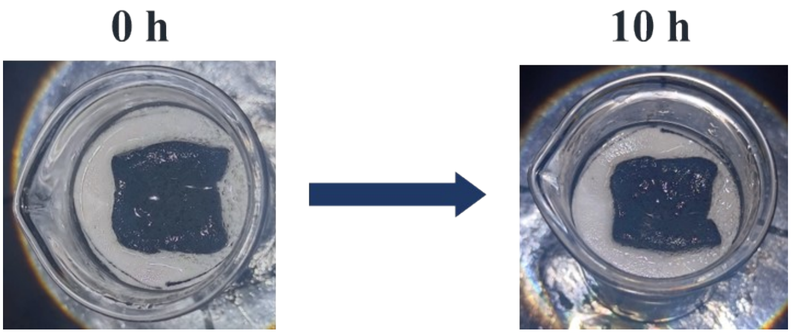

Figure S20. Digital photographic documentation of SiC@C/HPP aerogel surfaces pre- and post-10-hour exposure to 10 wt.% NaCl solution under continuous 1 sun illumination (1 kW·m^{-2}).

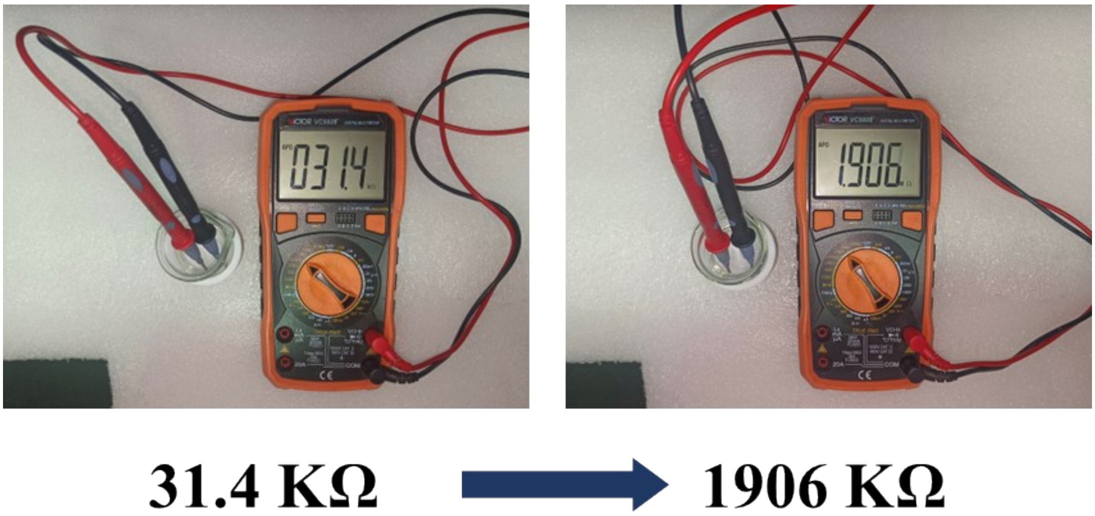

Figure S21.Resistance values of source seawater (left) versus distilled condensate (right).

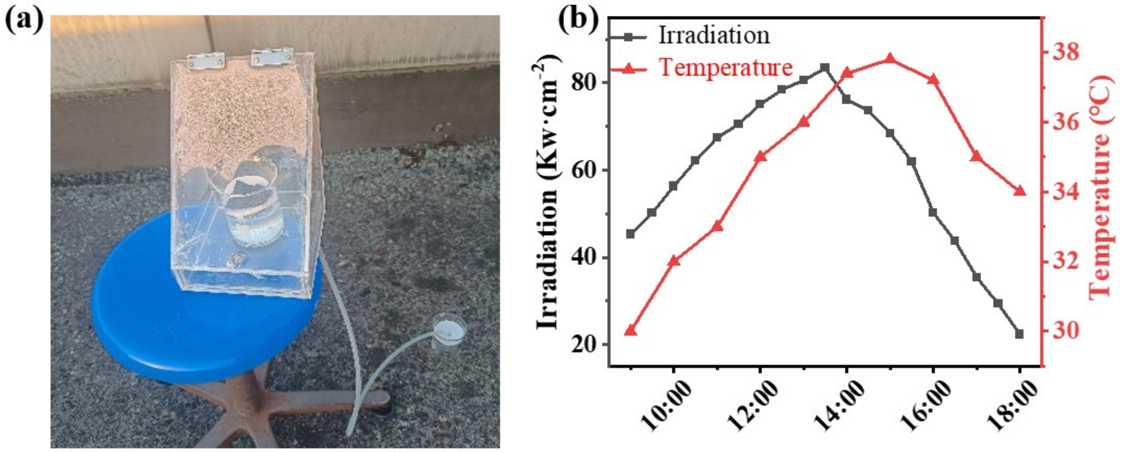

Figure S22. Outdoor interfacial solar evaporation performance validation: (a) Schematic of the experimental setup for natural sunlight-driven evaporation testing; (b) Temporal profiles of solar irradiance (black curve) and environmental temperature (red curve) recorded at 10-min intervals over 9 hours.

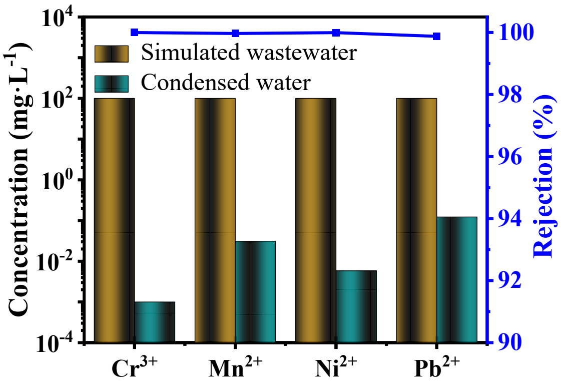

Figure S23. Concentrations and ion rejection rates (%) of the heavy-metal ions (Ni^{2+}, Pb^{2+}, Cr^{3+}, Mn^{2+}) in the simulated wastewater and the collected condensate.

Table S1. Comparative analysis of compressive mechanical properties between biomimetic SiC@C/HPP aerogels and representative aerogel systems reported in the literature.

Reference	Compressive strain (%)	Stress (Kpa)	Material
41	50	97.2	S@BC-CF33%-FD aerogel
42	70	55.6	Cellulose Nanofibril Aerogels
43	70	68	Cellulose-based aerogels from sugarcane bagasse
44	70	10	Graphitic carbon nanofiber aerogel
45	98	228.9	High-entropy ceramic aerogel
46	80	370	Silica sol-reinforced flexible silica aerogel
47	80	20	Poly(2dimethoxymethylsilyl)ethylmethylvinylsiloxane (PDEMSEMVS) aerogel
48	70	330	Novel salicylaldimine-bridged silica aerogel
49	90	237	Graphene aerogel
This study	70	523.1	SiC@C/HPP aerogel

Table S2. Benchmarking of water evaporation rates for biomimetic SiC@C/HPP aerogels against state-of-the-art photothermal evaporators under standardized 1 sun illumination (1 $kW \cdot m^{-2}$).

Reference	Evaporation rate ($kg \cdot m^{-2} \cdot h^{-1}$)	Material
50	2.18	Micro–nano water film enhanced interfacial solar evaporator
51	2.04	Sponge hydrogel evaporators (SHEs)
52	1.77	Hierarchical structured hydrogel evaporators (HSEs)
53	2.1	Plasmon based double-layer hydrogel
54	2.7	Double layer hydrogel evaporator
55	1.75	Bamboo leaf-derived carbon-based evaporator
56	2.51	UCOF-366-OH Double-layer interface water evaporator
57	2.096	Janus evaporator based on self-healing hydrogels
58	2.3	Ammonium dihydrogen phosphate-modified melamine sponge
59	2.48	Higher metal molybdenum nitride/melamine
This study	3.24	SiC@C/HPP aerogel

Table R1. Comparison of the biomimetic SiC@C/HPP aerogel with other representative solar evaporators reported in the literature.

Evaporator	Manufacturing Cost (USD/m²)	Ease of Fabrication	Evaporation Rate ($kg \cdot m^{-2} \cdot h^{-1}$)	Commercialization Potential
3D-printed bionic hydrogel evaporator (3DP-BHE)	$10.14	Moderate	2.13	Promising; 3D printing can be scaled for mass production
Biomass-derived Janus composite evaporator	$3.9	Simple	1.68	High; low cost and scalable fabrication via one-step immersion or spraying
UB-PP evaporator (pomelo peel + carbon black)	$2.7	Simple	1.81	High; both cost and method ideal for mass production
MOF-melanin hybrid evaporator (MNM)	$6.5	Relatively complex	1.34	Moderate; higher material cost but still promising for sustained outdoor use
PPy-coated polyester fiber sphere (PPy-PFS)	$25.97	Moderate	2.10	Moderate; scalable coating process, mass-production feasible
**This study**	**$25.59**	**Moderate**	**3.24**	**Moderate; raw materials are widely available, but freeze-casting still faces scale-up obstacles**
